# Smart Optogenetics for Real‐Time Automated Control of Cardiac Electrical Activity

**DOI:** 10.1002/advs.202522759

**Published:** 2026-02-13

**Authors:** Shanliang Deng, Niels Harlaar, Juan Zhang, Sven O. Dekker, Nina N. Kudryashova, Huiling Zhou, Cindy I. Bart, TianYi Jin, Georgy Derevyanko, Willem van Driel, Alexander V. Panfilov, René H. Poelma, Antoine A. F. de Vries, GuoQi Zhang, Tim De Coster, Daniël A. Pijnappels

**Affiliations:** ^1^ Laboratory of Experimental Cardiology Department of Cardiology Heart Lung Centre Leiden Leiden University Medical Center Leiden The Netherlands; ^2^ Department of Microelectronics Delft University of Technology Delft The Netherlands; ^3^ Department of Chemistry and Biochemistry Concordia University Montreal Quebec Canada

**Keywords:** cardiac arrhythmias, LED technology, machine learning, optogenetics, real‐time control loop

## Abstract

Control theory underpins the stabilization of dynamic systems, including cardiac tissue, where disruptions in electrical conduction cause arrhythmias. Current treatments either act rapidly but without precision or deliver targeted interventions that cannot adapt in real time. We present an integrated platform combining optical voltage mapping (OVM), machine learning (ML), and optogenetics for autonomous, real‐time detection and correction of cardiac rhythm disorders in vitro. OVM provides high‐resolution membrane potential visualization; the ML module identifies arrhythmic events and drives microLED‐based light patterns restoring normal conduction; and optogenetics enables light‐based modulation of excitable cells. This integration of electrical, optical, and bioelectrical domains through a unified computational control layer enables adaptive, closed‐loop rhythm stabilization, a significant advance in real‐time electrophysiological interventions. Because inference and actuation run in real time on modest hardware, the same control loop could be embedded into miniaturized devices or microcontrollers, accelerating the transition from in‐vitro to in‐vivo automated rhythm management.

## Graphical abstract

We are able to stop dangerous heart‐rhythm spirals before they fully form. Within about 100 ms, it pinpoints the spiral's tiny central tip (≈0.9 mm) using light‐based sensing and machine learning, then shines targeted light to shut it down. This fast, precise, closed‐loop approach detects, targets, and terminates arrhythmias in real time.

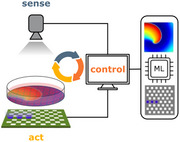

## 1 Introduction

Control theory is a foundational principle in engineering, enabling systems to self‐regulate by continuously adjusting their behavior in response to real‐time data [1]. These control loops operate across a wide range of temporal scales, i.e., from microsecond responses in electrical circuits [2] to long‐term adaptations in structural systems [3], each tailored to the dynamics of the process being managed.

In medicine, control mechanisms are equally critical, particularly when rapid responses are necessary to prevent severe or even fatal outcomes. Cardiac arrhythmias represent a prominent example, as abnormal heart rhythms typically require swift intervention due to their progressive and detrimental nature [4, 5, 6, 7]. Importantly, arrhythmias are not binary events but display diverse morphologies and propagation dynamics. Many arrhythmias are sustained by spiral waves that rotate around phase singularities (PSs) [8]. These PSs act as organizing centers and can drift across the tissue, creating a fundamental spatiotemporal challenge: how to reset such continuously changing behavior in a quick and accurate manner to restore normal heart rhythm. Ideally, anti‐arrhythmic strategies should therefore combine both speed and accuracy. Existing clinical approaches, however, often sacrifice one dimension for the other. Electrical cardioversion provides rapid but nonspecific termination by delivering high‐energy shocks indiscriminately to the entire heart. Catheter ablation offers precise targeting of stable rotors but lacks the ability to adapt in real time to their motion. Further improvement in anti‐arrhythmic therapy could therefore benefit from continuous monitoring of spiral wave activity and dynamic interventions that track and respond to the evolving position of PSs. Current clinical and experimental platforms, however, lack this level of adaptive control for bioelectrical dynamics at spatiotemporal scales needed in cardiac tissue.

At the single‐cell level, real‐time optogenetic feedback has been achieved using dynamic patch‐clamp techniques [9, 10]. Extending this concept to the tissue scale, previous studies have demonstrated that real‐time closed‐loop optogenetic control of cardiac dynamics is feasible from a delay‐based perspective, both in 1D settings that rely solely on arrival time [11] and in 2D settings that explicitly account for wave propagation and spatiotemporal dynamics [12]. In parallel, advances in LED matrix technology have enabled patterned optical stimulation for optogenetic control in non–real‐time [13] and, in work conducted concurrently with ours (though still at the preprint stage), in real‐time implementations as well [12].

Initial insight into how to optimally terminate reentrant arrhythmias and preemptively suppress their formation can be gained by studying the rapid spatiotemporal dynamics of rotor initiation and extinction in vitro. To enable this, we developed a specialized platform for probing and manipulating the onset and termination of reentrant electrical waves in cardiac tissue with high spatial and temporal resolution. This system combines and builds upon in‐house expertise in optical mapping [14, 15], machine learning (ML)‐driven signal analysis [16], and precision optogenetic stimulation [17, 18] to form a closed‐loop control platform capable of real‐time adaptive intervention [10]. The platform operates in three integrated stages. First, ML algorithms (the detector) analyze cardiac electrical signals in real time to identify arrhythmic events. Second, the system determines the optimal parameters for effective light stimulation. Third, matrices of mini‐light‐emitting diodes (mLEDs) deliver targeted and patterned optogenetic pulses to terminate simple or complex reentries within a single well or simultaneously across different wells.

By bridging the gap between observation and intervention, our integrated system offers a powerful platform for exploring fundamental questions about rotor dynamics, optimal termination strategies, and feedback‐driven rhythm stabilization. It is a key step toward shock‐free, intelligent arrhythmia therapies rooted in real‐time bioelectronic control, reducing tissue damage and alleviating pain.

## 2 Results

### 2.1 System Overview

**FIGURE 1:**
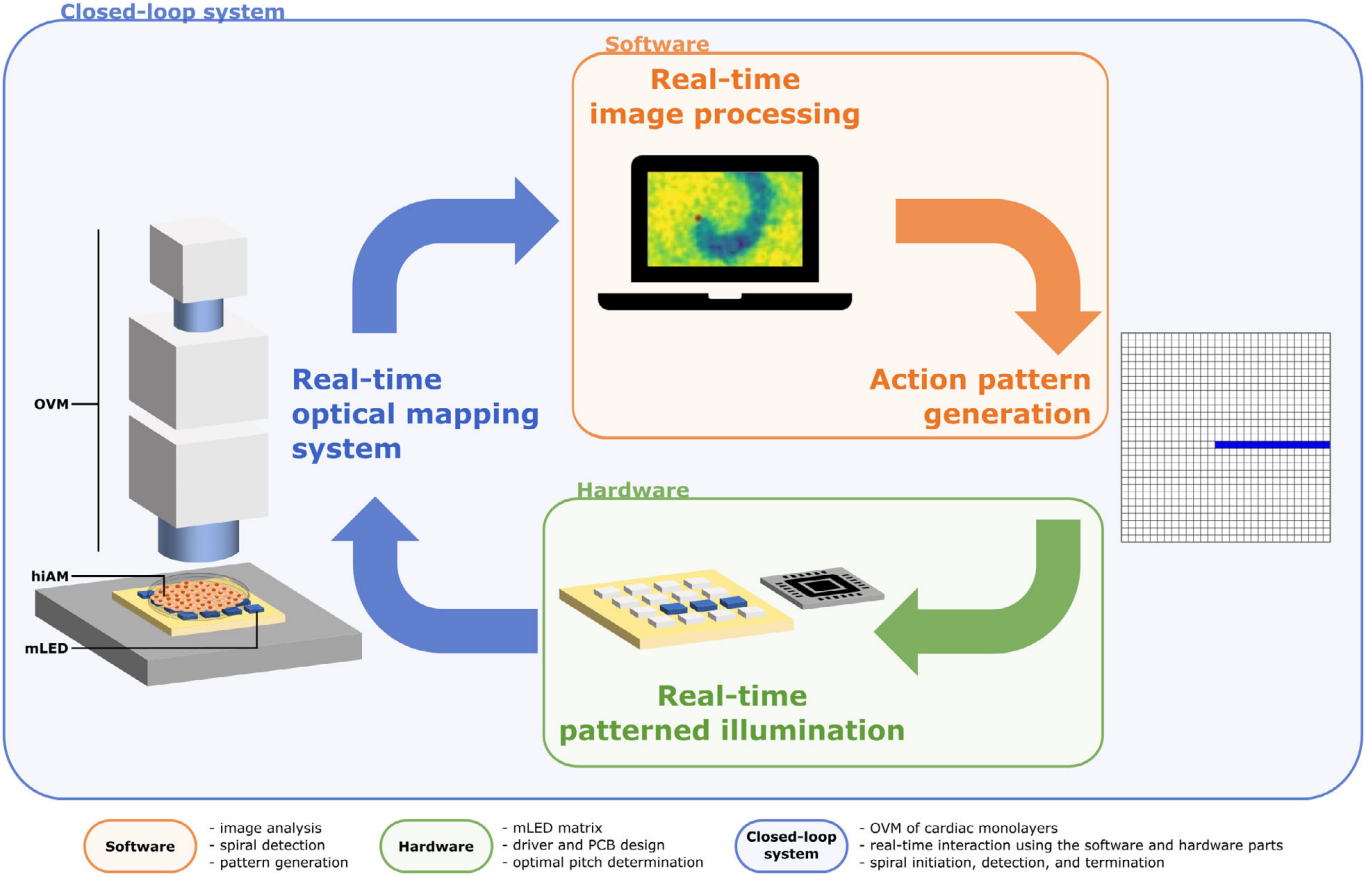
Schematic overview of the integrated software and hardware platform (system) for real‐time optical voltage mapping (OVM) and manipulation of cardiomyocyte (hiAM) monolayers. The system is divided in three main components: software (orange background) for real‐time image processing, AI‐driven spiral wave detection, and adaptive action pattern generation; hardware (green background) featuring an mLED matrix with custom driver circuitry, PCB design, and optimized spatial pitch for precise pattern illumination; and a closed‐loop interface (blue background) that synchronizes real‐time optical data acquisition with targeted illumination, enabling automated initiation, detection, and termination of spiral waves. This bidirectional system provides rapid feedback for investigating arrhythmia dynamics and intervention strategies.

To enable simultaneous monitoring and control of cardiac electrical activity in a closed loop, we designed a customized optical system (Figure 1) that integrates a standard optical voltage mapping (OVM) setup with tailored optogenetic stimulation and intervention (Figure 1), blue background). This is achieved by adding two layers to our setup: hardware (Figure 1), green background) and software (Figure 1), orange background) for real‐time image processing and patterned illumination. This tripartite color coding is used throughout the manuscript.

This three‐component platform enabled real‐time recording of membrane voltage dynamics while precisely manipulating cellular behavior.

The *first component* comprises human conditionally immortalized atrial myocytes (hiAMs) chosen for their relevance to atrial arrhythmias and capacity to sustain high‐frequency reentrant circuits [19]. These hiAMs were engineered with the blue light‐sensitive channelrhodopsin CheRiff [20] (Figure S1) to enable optogenetic, shock‐free [21] control of their electrical activity (see Methods 4.1.1) while avoiding spectral overlap with the excitation and emission spectrum of the voltage‐sensitive dye Di‐4‐ANBDQBS (Figure S2b red and orange lines). The resulting cell line was designated CheRiff‐hiAM. As a *second component* of our closed‐loop platform (hardware), an mLED matrix was positioned beneath the culture plate to enable targeted illumination of specific regions within the cell monolayers (Figure S2a).

**FIGURE 2:**
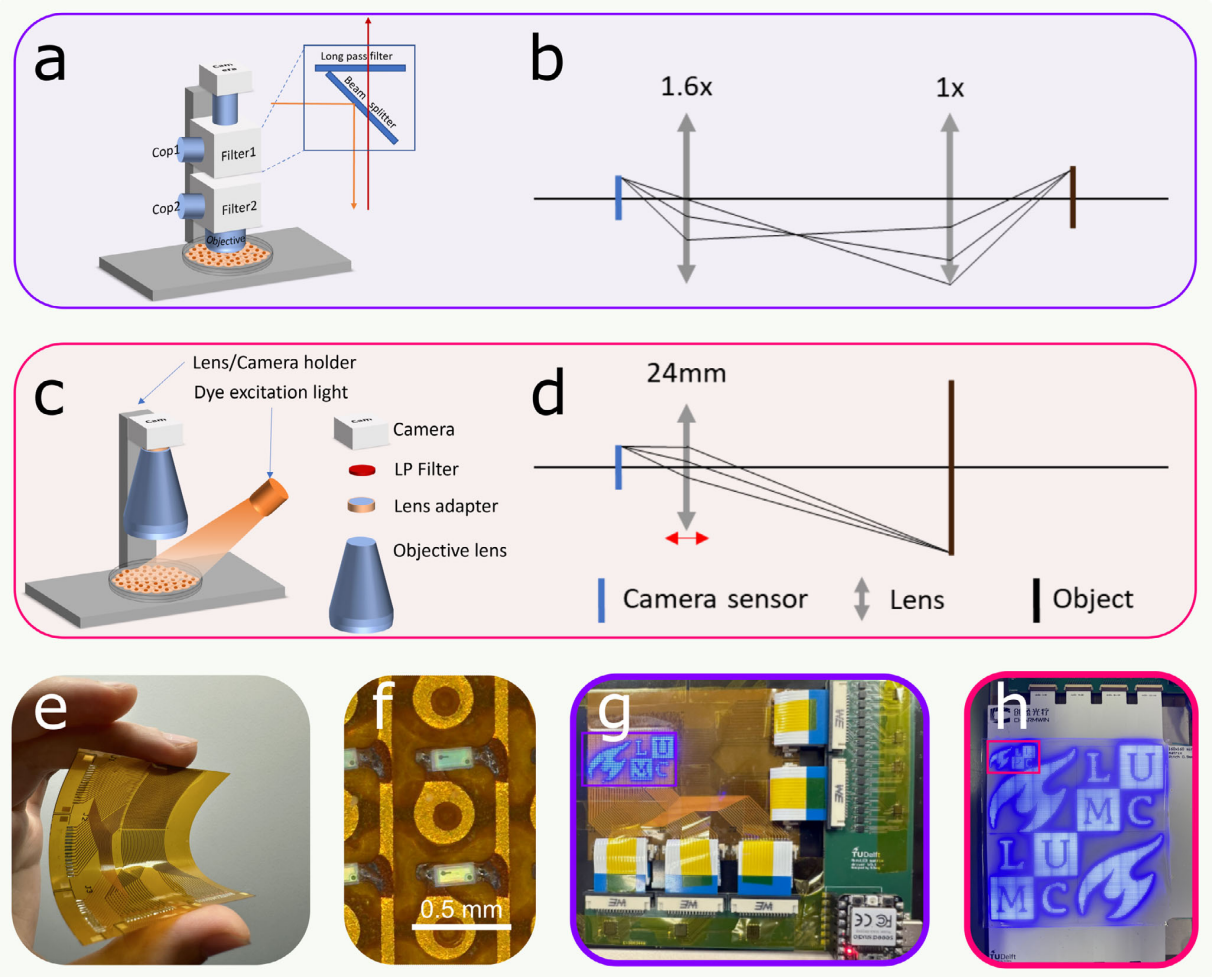
Hardware design of the closed‐loop imaging and mapping components. (a–d) OVM hardware. (a) Configuration of the narrow field‐of‐view OVM system, including a long‐pass (LP) filter (Filter), collimating mirror (Cop), heating plate, cell monolayer, and camera. (b) Optical pathway for the setup in (a), showing the condenser lens (left 1.6×) and objective lens (right 1×). (c) Configuration of the wide field‐of‐view mapping system, including a lens adapter with a long‐pass filter, side illumination for excitation of the potentiometric dye, heating plate, cell monolayer, and camera. (d) Optical pathway for the setup in (c), featuring a 24 mm objective lens. (e–h) mLED matrix hardware. (e) Flexibility of the small‐sized mLED matrix. (f) Close‐up of individual pixels/LEDs in the small‐size mLED matrix. (g) Fully assembled small‐size mLED matrix (48 × 32 pixels) displaying the TU Delft and LUMC logos. (h) Fully assembled large‐size mLED matrix (160 × 160 pixels) displaying the same logos with the red‐outlined region indicating the size of the small mLED matrix.

To accommodate different monolayer formats, we developed two configurations of the mLED matrix and OVM setup (Figure 2). The first configuration (Figure 2a; Figure S2a, left) combines a small mLED matrix with a dual‐lens optical path (Figure 2b), optimized for high‐resolution imaging of small CheRiff‐hiAM layers (e.g. wells of a 12‐well plate), with the field of view (FOV) limited by the magnification of the lenses. The second configuration (Figure 2c,d; Figure S2a, right) employs a larger mLED matrix and a commercial camera lens, providing a significantly larger FOV suitable for extended monolayer formats up to 14 cm in diameter, enabling broader experimental applications.

The *third component* of our closed‐loop platform is a software layer that integrates three key functions into a single user‐friendly program. Initially, images of the cell monolayer (first component) are rapidly acquired via a direct connection with the camera. Next, intelligent image analysis predicts the spiral cores of reentrant waves. Based on this prediction, a precise stimulation pattern is generated and sent to the mLED matrix (second component) to terminate the reentrant wave.

The software interfaces the hardware and the tissue, closing the bio‐opto‐electronic control loop for precise, intelligent, and shock‐free control of heart rhythms. Detailed descriptions of each component and selected biomedical applications follow.

### 2.2 Hardware: MLED Matrix

Light‐based technologies are increasingly employed to investigate and manipulate biological systems due to their unparalleled spatiotemporal precision [22, 23]. Optical stimulation enables targeted activation or inhibition of biological processes with high resolution, which requires light delivery systems that are both accurate and compatible with biological contexts. Recent advances in LED technology have facilitated the development of LED matrices that meet the critical demands of biomedical research: miniaturization, scalability, flexibility, and biocompatibility [24, 25, 26]. In this context, we developed two custom addressable LED arrays optimized for patterned in vitro illumination, enabling precise and dynamic control of light exposure in cardiac monolayer experiments.

#### 2.2.1 mLED Matrix Design

The first mLED matrix was fabricated on a flexible polyimide substrate (Figure 2e). A zoomed‐in view of the individual mLEDs (Figure 2f) shows their dimensions of 125 × 250 µm^2^. Two versions of the matrix were designed and fabricated to match the OVM configurations described above. The small matrix, consisting of 48 × 32 pixels with a resolution of 0.6 mm, is suitable for 12‐well monolayers (Figure 2g). The larger matrix, designed for the large FOV setup, has 160 × 160 pixels with an optimized resolution of 0.9 mm (Figure 2h). The mLED matrices were designed to reach ∼80% light uniformity (Figure S3, see Methods 4.2.3) and produced only a minimal temperature increase under the termination conditions that were used, remaining well below levels that could damage cardiac tissue (Figure S4, see Methods 4.2.4). The light intensity reached a maximum of 116 mW/cm^2^ for the small matrix and 65.4 mW/cm^2^ for the large one (Figure S5a), with both matrices having a center wavelength of 450 nm.

#### 2.2.2 Optogenetic Manipulation Validation

To validate its function, basic optogenetic manipulation tests were performed using the small mLED matrix on ≈3.8 cm^2^ CheRiff‐hiAM monolayers (single wells of a 12‐well plate).

##### 2.2.2.1 Optical Pacing

First, optical pacing was performed across the bulk of the monolayer (Figure S5b, see Methods 4.1.2) to confirm its excitability. We then investigated how the number of optical pulses required for four consecutive successful inductions of action potentials varied with the number of mLEDs used (Figure S5c). The monolayer could be paced with 100% success (i.e., each of the four optical pulses created a propagating wave of excitation) using a single mLED at 30 mW cm^−2^, while four mLEDs in a square pattern achieved full capture at 10 mW cm^−2^. Activation maps of the optically triggered activity confirmed that the origin of activation coincided with the illuminated area (Figure S5d).

##### 2.2.2.2 Conduction Block

Given that blue light depolarizes CheRiff‐hiAMs, we tried to create a line of functional conduction block using the mLED matrix (Figure S6a,c) to stop or deflect incoming paced waves. At low light intensity, the excitation wave still passed through the illuminated area (Figure S6a,b), but at higher intensity, propagation was fully blocked (Figure S6c,d). The efficiency of the conduction block depended on both line thickness and light intensity (Figure S6e). With a single line at >40 mW cm^−2^, all incoming pacing waves were blocked, demonstrating the mLED matrix's capability to terminate reentrant waves optogenetically.

##### 2.2.2.3 Reentry Induction and Termination

To further investigate the capability of the mLED matrix to terminate reentrant waves, we used an S1S2 protocol to induce reentrant waves (Figure S7a–c). By adjusting the interval between the electrical S1 and optical S2 pulses, reentry was reproducibly induced, with one example shown in Figure S7d. To terminate reentry, we manually applied a single 0.5 s light line once the reentrant wave had stabilized, connecting its core to the nearest inexcitable border (Figure S7e,f). After termination of the re‐entry, sinus rhythm was restored by electrical pacing. These experiments demonstrate the system's capacity to precisely manipulate cardiac electrical activity with light.

#### 2.2.3 Optimization of the mLED Matrix

A major advantage of patterned over global illumination for terminating reentry is improved power efficiency. The spatial arrangement of the mLEDs within the light line affects the total power required to terminate reentrant waves. To determine the minimum power needed for termination, we tested five line patterns with pixel pitches ranging from 0.3 to 1.68 mm (Figure S8a). Pixel pitches between 0.8 and 0.9 mm proved optimal, guiding the design of the large mLED matrix (Figure S8b,c; Figure S9).

### 2.3 Software: Spiral Wave Core Detection

Deep learning is increasingly applied in biomedical research but typically requires large, annotated clinical datasets, limiting its use to areas where such data are available, e.g., ECG analysis [27]. A strategy to address data scarcity is synthetic data generation. This approach to data augmentation is widely used in engineering applications [28], such as training of self‐driving cars [29] or robotics [30]. However, in biomedical research, synthetic data generation is particularly difficult [31], as it requires realistic models that capture the complexity of biological systems.

One of the areas in biomedicine where computational modelling has seen a steep rise in popularity and accuracy is cardiology [32]. The rich physiological datasets from arrhythmia studies have enabled the construction of detailed mathematical models that capture the complex dynamics of cardiac rhythm [33, 34]. These models are able to produce realistic wave propagation patterns and have revealed new insights into the mechanisms underlying cardiac arrhythmias [35, 36, 37]. A key finding is that arrhythmias often consist of spiral waves rotating around PSs [8]. Identifying PSs is central to clinical diagnosis and treatment [38], yet experimental recordings typically lack sufficient spatial resolution to do so reliably [39]. Mathematical models can overcome this limitation, thereby enabling the generation of realistic, annotated synthetic datasets with precisely located arrhythmia sources.

#### 2.3.1 Convolutional Neural Network (CNN)

**FIGURE 3:**
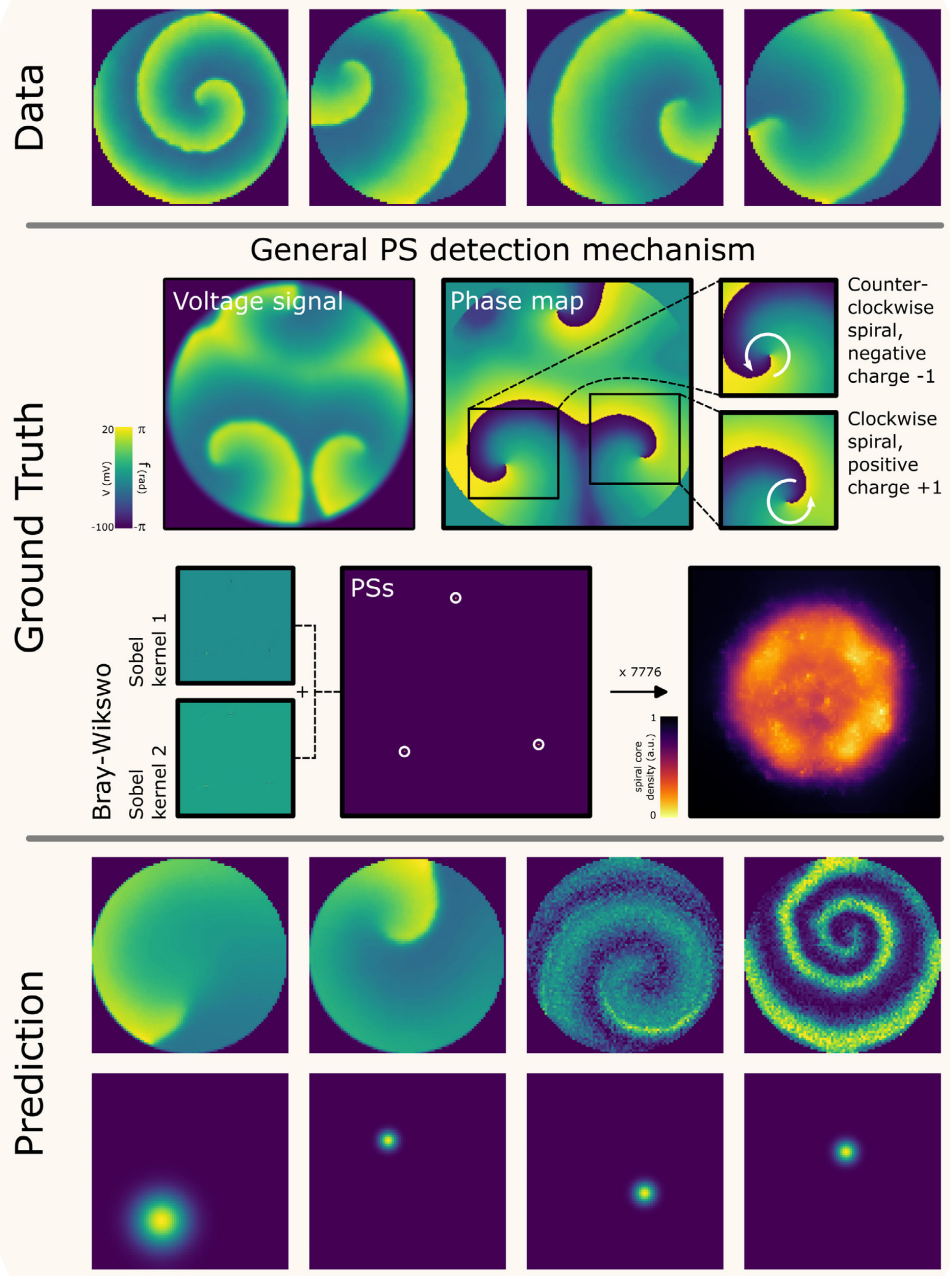
Software design for spiral core detection. A CNN was developed to identify spiral cores. Training data (top) was created in silico (for details, see Figure S10), and augmented by image flipping and rotation. Ground truth (middle) spiral core locations were determined using the Bray–Wikswo phase singularity (PS) detection algorithm, with the corresponding density distribution shown on the right. After training, the CNN produced accurate predictions (bottom), precisely localizing spiral wave cores.

To identify cardiac arrhythmias by locating spiral wave PSs, we trained a shallow CNN, selected for its ability to efficiently capture spatially localized features while maintaining computational efficiency, using synthetic data from a computer model (see Materials and Methods) that accurately reproduces cardiac conduction in monolayers and incorporates heterogeneity (Figure S10a, left). Single spiral waves were initiated using an S1S2 protocol, after which the properties of each sample, such as the wave propagation speed, action potential duration, and spiral frequency, were measured (Figure S10a, right). Starting from these spirals, more complex spiral activity could also be generated (Figure S10b). To evenly distribute spiral core locations (all spirals initially rotated in the same direction), the data were flipped and rotated (Figure 3, top). For each spiral, the ground truth core position was calculated using the Bray–Wikswo algorithm [40] (Figure 3, middle), producing a homogeneous distribution across virtual monolayers (Figure 3, middle). These ground truth labels, combined with the flipped and rotated spirals, were used to train the convolutional neural network (Figure S11a,b). The training objective was to identify the centers of spiral waves from limited temporal data, comprising less than a single rotation (30 ms of activity). The resulting algorithm accurately predicted spiral wave centers, including in noisy data (Figure 3, bottom). Validation on previously recorded cardiac monolayer data further confirmed its accuracy (not shown).

#### 2.3.2 Different Cell Types

The algorithm was trained with synthetic data for neonatal rat ventricular myocytes for two practical reasons: (1) availability of experimental recordings [41, 42] to verify the algorithm, and (2) existence of an accurate mathematical model capable of realistically replicating monolayers of these cells [43], allowing generation of synthetic training data. All training and validation used archived data from our in‐house optical mapping database.

For translational purposes, all new experiments were conducted on optogenetically modified hiAMs [19]. Remarkably, the trained algorithm accurately identified spiral wave PSs in monolayers of these human cells without needing transfer learning. This demonstrates that our shallow convolutional neural network captures spiral wave dynamics independent of species (rat/human), heart chamber (ventricle/atrium), and action potential shape and duration (Figure S12).

### 2.4 Closed‐Loop Feedback: Integration of Hardware, Software, and Biology

To maximize the speed and reliability of reentrant wave termination, we developed a fully automated system that integrates the mLED matrix with our machine learning‐based spiral wave core detection algorithm, enabling rapid and reliable closed‐loop feedback (Figure S13).

#### 2.4.1 Real‐Time Automatic Reentry Termination

**FIGURE 4:**
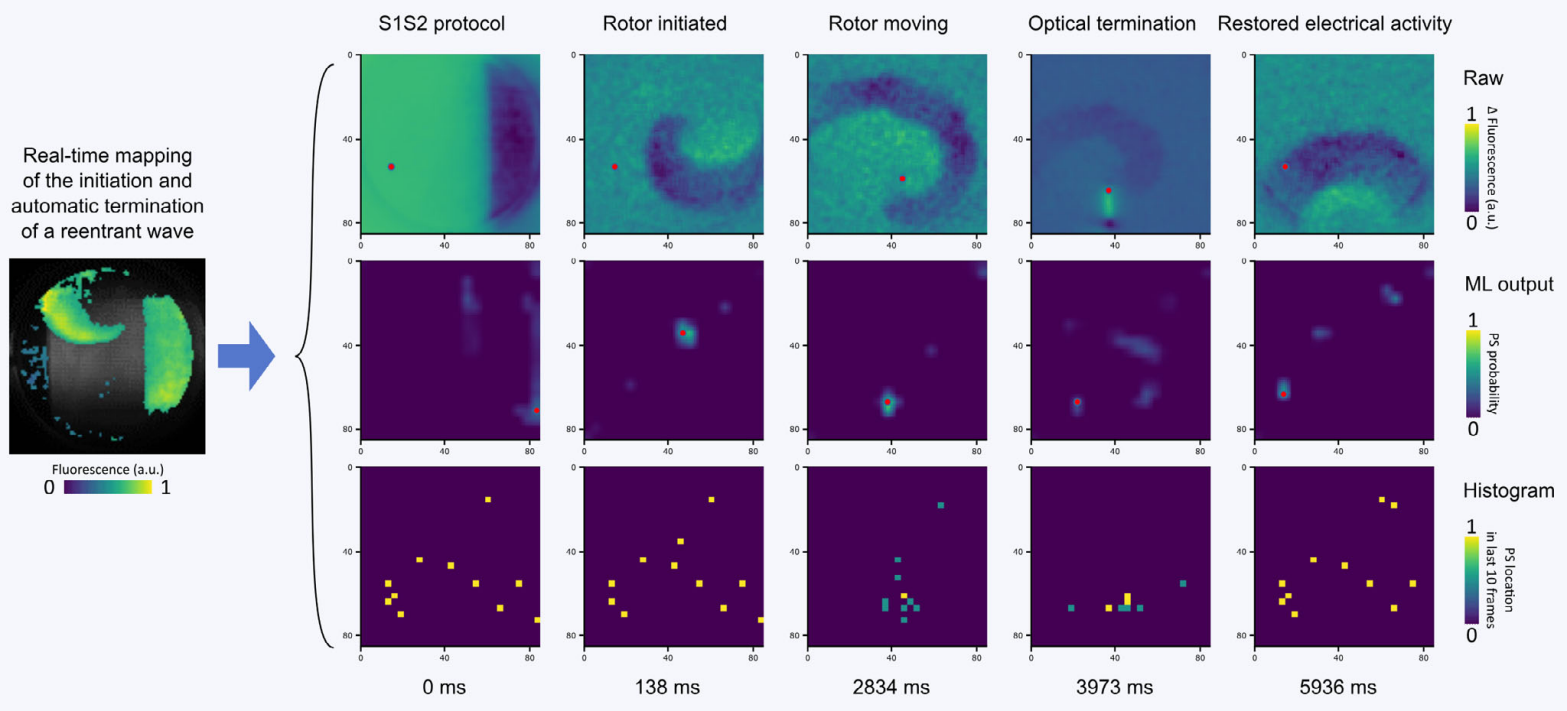
Real‐time closed‐loop mapping of programmed initiation and automatic termination of reentrant waves in cardiac monolayers. Three sets of images were acquired with the dual‐lens OVM setup and the small mLED matrix (Figure 2A,G) and subsequently analyzed using our custom software, as illustrated for an experiment in a 12‐well cardiac monolayer: (1) Top row: Raw images at different stages of reentry wave progression: S1S2 protocol, rotor initiated, rotor moving, optical termination, and restoration of sinus rhythm. (2) Middle row: machine learning (ML)‐generated maps showing the predicted spiral core locations with probabilities ranging from 0 to 1. (3) Bottom row: Histogram arrays tracking the most probable core positions over the previous 10 frames, with the current core marked as a red dot in the raw images.

From induction to successful termination of reentry (Video SV1), the system workflow comprises five consecutive steps (Figure 4, columns) across three layers of information (Figure 4, rows). Raw images from the OVM camera were first preprocessed (Figure 4, top row) and then passed to the core detection algorithm, which produced a probability map of spiral core locations with scores ranging from 0 to 1 (Figure 4, middle row). The pixel with the highest probability was tracked over time using a histogram to determine the most consistent core location (Figure 4, bottom row). This location was marked on the raw image as a red dot. When the average core probability score exceeded 0.7 for 10 consecutive frames, an optical stimulation line was automatically generated, connecting the detected core to the nearest boundary.

Localization error relative to the LED pitch is not a limiting factor in our system, as the camera provides higher spatial resolution than the mLED matrix. Predictions are therefore generated at this finer resolution and subsequently downsampled to ensure coverage of the PS. Frame‐by‐frame detection accuracy is influenced primarily by biological noise levels and cannot be quantified precisely without a ground‐truth reference. Nonetheless, prior work has shown that PS localization accuracy deteriorates as the standard deviation of additive Gaussian noise increases [48], consistent with our observation that high‐noise conditions produce to unstable detections (mitigated in practice by the histogram‐based stabilization). More importantly, overall system performance is constrained by tolerance to spatial misalignment between the camera, detection algorithm, and mLED matrix. Experimentally, this tolerance was determined to be 1.8 mm (three LEDs for the small matrix, tested for n = 3 monolayers, Figure S14).

**FIGURE 5:**
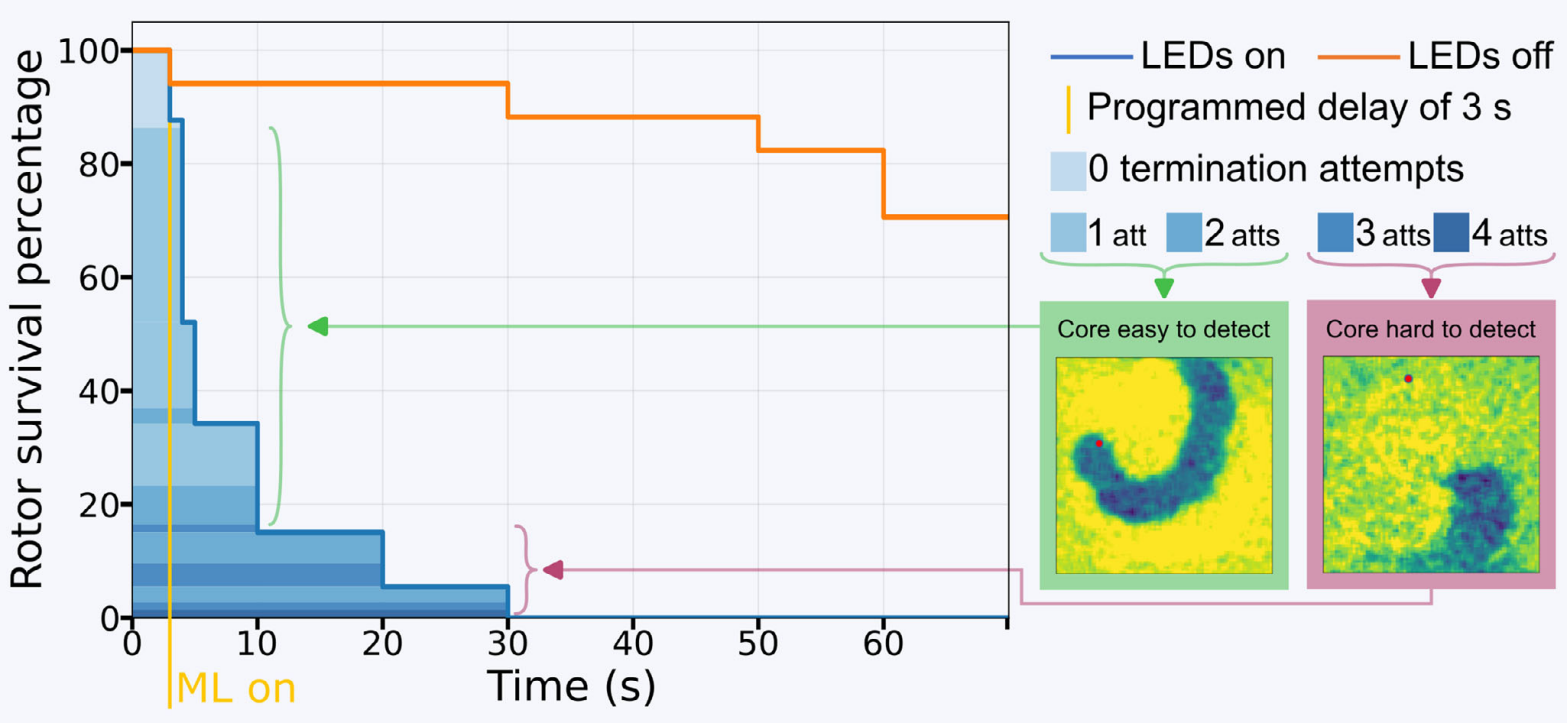
Rotor survival percentage with and without real‐time closed‐loop feedback. When machine learning (ML) is not active (orange line), rotors exhibit prolonged survival, persisting beyond 60 s. In contrast, activation of real‐time feedback (blue line) leads to rapid rotor termination within 5 s. A qualitative relationship was found between termination attempts, termination time, and the degree of mathematical spiral wave similarity. Experiments were performed on monolayers in a 12‐well plate format using the dual‐lens OVM setup and the small mLED matrix (Figure 2A,G).

Despite core migration in some monolayer cultures, the system reliably tracked the cores and efficiently terminated reentries across 73 events in five samples (Figure 5). A 3 s programmed delay was applied, allowing the spiral waves to settle. During this period, approximately 10% of spiral waves terminated spontaneously. Within the following 2 s, approximately 50% of spirals were terminated with a single termination attempt. Over 80 % of the reentrant waves were terminated within 10 s and using less than three termination attempts. All reentry waves in the experimental group were terminated within 30 s and maximally needing 4 termination attempts (see Figure S15 for the most common failure patterns). In contrast, in the no‐light control group (17 events in two samples) termination occurred in <10% of cases at 30 s, rising to 30% after 1 min. Reentries with non‐classical spiral morphology (Figure 5, red border) required longer recognition times and/or more termination attempts.

#### 2.4.2 Advanced Real‐Time Automatic Reentry Termination

**FIGURE 6:**
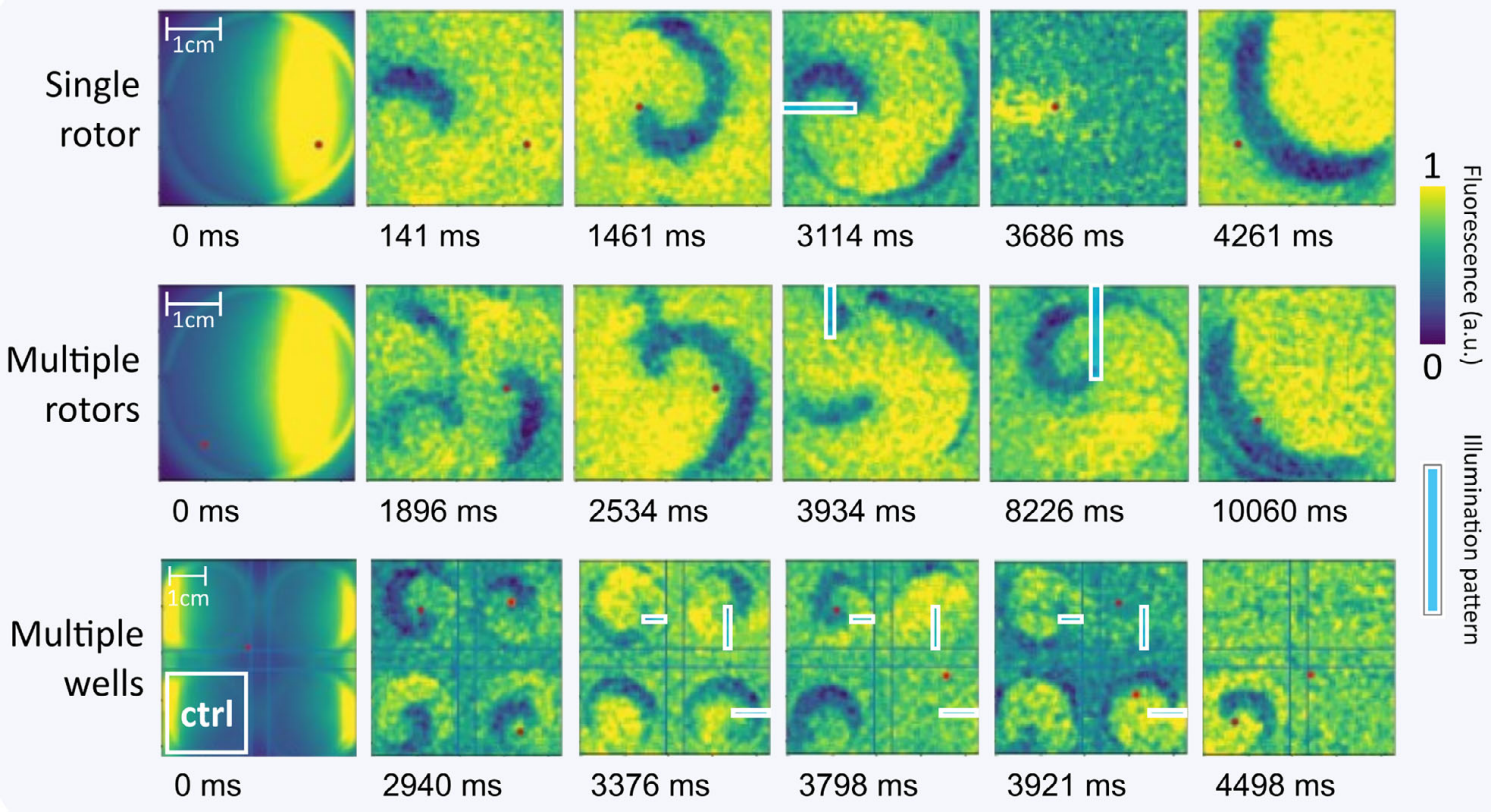
Multi‐rotor and high‐throughput termination capabilities of the automatic reentry termination feedback loop. Three representative use cases are shown. (1) Top row: Time‐lapse images showing the initiation and termination of a single rotor in a 6‐well plate using the large field‐of‐view OVM setup and large‐size mLED matrix. (2) Middle row: Time‐lapse images demonstrating the system's ability to localize and terminate multiple reentry waves within a 6‐well monolayer. (3) Bottom row: Time‐lapse images illustrating high‐throughput operation across four 12‐well monolayers, with the bottom‐left well serving as a control well in which the LED output was turned off.

In atrial fibrillation, multiple reentrant waves often occur in the atrium. To explore advanced, automated reentry termination under such conditions, we employed the large FOV OVM setup (Figure 2C) with the large mLED matrix (Figure 2H) to test the system in single wells of a 6‐well plate (≈9.6 cm^2^) as well as across multiple wells of a 12‐well plate (Figure 6; Figure S16).

As an initial validation, a single reentrant wave was induced in a 9.6 cm^2^ CheRiff‐hiAM monolayer using an optical S1S2 protocol. The system rapidly located the spiral core and terminated the reentry by generating a line of light connecting the core to the nearest border within 114 ms after the programmed 3 s delay (Figure 6, top row). To evaluate the system's capacity for handling multiple rotors, we induced several reentrant waves within a single CheRiff‐hiAM monolayer in a 6‐well plate. After stabilization, the algorithm located the dominant rotor and terminated it via targeted optical stimulation. The remaining rotor was subsequently terminated using a second optical line (Figure 6; middle row and Video SV2). To assess the system's higher‐throughput capability, reentrant waves were induced in four adjacent CheRiff‐hiAM monolayers in a 12‐well plate. The LED output for the bottom‐left well was disabled to verify that illumination in one well did not affect reentry in neighboring wells. The spiral wave cores in the three “active” wells were located within 376 ms, and optical lines were generated simultaneously to terminate the reentrant waves. A single light pulse successfully terminated the reentrant waves in the “active” wells, while reentry persisted in the control well (Figure 6 bottom row; Video SV3).

## 3 Conclusion

In this manuscript, we introduce a novel closed‐loop control platform that integrates optical mapping, machine learning‐based signal analysis, and precise optogenetic stimulation to perform real‐time adaptive interventions in cardiac tissue. This system enables dynamic probing and manipulation of reentrant electrical wave activity with high spatiotemporal resolution. Its key innovation lies in adaptivity: the platform continuously monitors irregular activity patterns in real time and automatically adjusts its intervention, effectively tracking the spatiotemporal evolution of spiral waves and dynamically relocating the site for optogenetic termination. Notably, the system executes these adjustments within approximately 100 ms at 0.9 mm spatial resolution, i.e. faster than one rotational period of a spiral wave and smaller than spiral core sizes in atria (1–5 mmm) and ventricles (3–10 mm). Furthermore, by employing optogenetics rather than electrical shocks to terminate arrhythmias, this approach is shock‐free, which is particularly promising for translational applications where shock‐induced pain remains a significant barrier to patient comfort and therapy compliance [43].

Our proof‐of‐concept study demonstrates that our closed‐loop control platform possesses strong potential but requires further refinement to enhance its robustness and translational potential. Currently, CheRiff serves as the optogenetic actuator; while effective in our hiAM cell model [19], newer, more light‐sensitive channelrhodopsins [44] such as bReaChES [45], ChRmine [46], or ChReef [47] could reduce light requirements, supporting long‐term or implantable applications where light delivery is constrained, e.g. for safety reasons. On the computational side, we employed a CNN, which performed well overall but was challenged under high‐noise conditions, consistent with prior in silico reports [48]. Alternative CNN architectures [48, 49] or transformer‐based approaches [50, 51], could further enhance accuracy, speed, and noise tolerance. Incorporating greater biological variability, such as irregular monolayer shapes or deformed spiral geometries, into training datasets may also improve model generalizability. Additionally, including scroll waves in 3D tissue engineering samples could provide further improvements.

Another area for improvement is the fixed capture rate, currently set at 8 ms. Under certain conditions, particularly when the spiral wave frequency aligns with the capture interval, the system may repeatedly encounter similar signal patterns, potentially leading to misclassification or detection delays. Implementing an adaptive capture rate could mitigate this risk, further enhancing reliable recognition of dynamic wave patterns. We also observed recurring patterns in the histogram‐based image analysis during periods of electrical quiescence, caused by light reflections from the optical setup. Optimizing the optical configuration or applying advanced artifact rejection algorithms [52, 53] could further improve the system's accuracy and specificity.

Looking ahead, our system provides a foundational research tool with strong potential to inform the development of future implantable devices. For clinical translation, the rigid components of the current prototype would need to be replaced with miniaturized, stretchable, and foldable light delivery structures [54, 55, 56]. Although we demonstrated feasibility using a flexible substrate, a stretchable mLED matrix would be better suited for conformal integration with cardiac tissue [57]. Furthermore, OVM is not suitable for in vivo use, necessitating a transition to electrode‐based systems [58]. We propose combining a minimally invasive electrode array (such as a micro‐electrode (implantable) array, or ME(I)A [59, 60, 61, 62]) with an integrated LED matrix. Such a hybrid device could enable continuous electrical monitoring and adaptive modulation, not only at the cardiac surface but also, via transmural access, in deeper layers of cardiac tissue. Achieving this would require substantial updates to the machine learning system, shifting from 2D signal interpretation to fully 3D modeling of cardiac electrical activity. This could be achieved either through direct 3D measurements from transmural arrays or by training neural networks to infer volumetric activation patterns from surface data alone [63, 64].

In the long term, integrating reinforcement learning into the machine learning component could enable the system to autonomously refine its intervention strategies [65, 66, 67], identifying high‐priority regions for stimulation and optimizing stimulation patterns under energy constraints. Such autonomous learning would be instrumental in identifying protocols for terminating arrhythmias using minimal energy, which is important for implantable or wearable applications. As machine learning models become more sophisticated while remaining compact (our current 2D model is only 62 kilobytes), they could be deployed directly on‐chip [68], paving the way for fully embedded, low‐power processing in wearable therapeutic devices. Because inference and actuation could run in real‐time on such modest hardware, our control loop could be embedded into miniaturized devices or microcontrollers, accelerating the transition from in‐vitro to in‐vivo automated rhythm management. Together, these advancements will support the development of next‐generation smart cardiac interfaces, enabling long‐term, adaptive, and patient‐specific arrhythmia management.

## 4 Experimental Section/Methods

### 4.1 Biomedical Methods

#### 4.1.1 OVM of Monolayers of CheRiff‐Expressing hiAMs

Two hiAM clones (2.38 and 2.86) were genetically modified with a self‐inactivating lentiviral vector (LV.HsUBC.CheRiff∼eGFP.IRES.PurR.hHBVPRE) encoding the depolarizing light‐gated ion channel CheRiff [20] fused to enhanced green fluorescent protein (Figure S1), and subsequently selected with puromycin to generate stable cell lines. OVM was then used to visualize the electrical activity of these CheRiff‐expressing monolayers during light stimulation. The monolayers were loaded with Di‐4‐ANBDQBS (AAT Bioquest, Pleasanton, CA) dye at 33 µM concentration for 15 min at 37°C. The dye‐containing medium was subsequently replaced by pre‐warmed DMEM/F‐12 (2 and 1 mL for wells of 6‐ and 12‐well plates, respectively) for OVM. Only monolayers showing uniform action potential propagation upon 1‐Hz electrical or optical pacing were further used (n = 7). When action potential durations were too long to induce reentry, the selective K_ATP_ channel opener P1075 was added to the culture medium at a final concentration of 10 µM to enable induction of reentrant activity.

#### 4.1.2 Stimulation Protocol

##### 4.1.2.1 Electrical Pacing

To verify homogeneous conduction in the CheRiff‐hiAM monolayers, six electrical pulses were delivered at 1 Hz through an epoxy‐coated bipolar platinum electrode delivering square 10 ms, 8 V suprathreshold electrical impulses via an STG 2004 stimulus generator and MC Stimulus II software (both from Multi Channel Systems, Reutlingen, Germany).

##### 4.1.2.2 Optical Pacing

To optically pace CheRiff‐hiAM monolayers, a dotted light pattern consisting of 1–4 LEDs was used (Figure S5a). In each optical pacing experiment, five light pulses each lasting 50 ms were delivered at 1 Hz frequency. The pacing threshold was determined by stepwise variation of the light intensity from 50 to 0 mW cm^−2^ (Figure S5b).

##### 4.1.2.3 Conduction Block

To optically block electrical wave propagation in CheRiff‐hiAM monolayers, an optical line 1–4 pixels wide and spanning the entire monolayer was applied after the first and up to the fourth electrical pacing pulse (Figure S6a–d). The conduction block efficiency was assessed by stepwise variation of the light intensity from 50 to 0 mW cm^−2^ (Figure S6e).

##### 4.1.2.4 S1S2 Protocol

Reentry was induced using a programmed optical stimulation (S1S2 protocol, Figure S7a–c) via the mLED matrix. An initial pulse was delivered at the edge of the monolayer, followed by a delayed light pulse covering half of the monolayer, thereby initiating reentry. The S1‐S2 interval was determined individually for each monolayer based on the action potential duration and conduction velocity [15].

#### 4.1.3 Experimental Protocols

To perform real‐time automated reentry termination experiments in 12‐well format hiAM monolayers, we employed a high‐resolution mapping setup together with a small (48 × 32) mLED matrix. The mLED matrix output remained disabled until a reentrant wave was induced. Reentry was triggered manually using an S1S2 pacing protocol (Section 4.1.2.4). After initiation of irregular wave propagation, the system waited 3 s to allow the reentry to stabilize. If a reentrant wave was not induced or auto‐terminated during this period, the S1S2 protocol was repeated. After the 3 s delay, the mLED matrix output was automatically enabled. Once the machine learning algorithm identified the spiral core and generated a line pattern for arrhythmia termination, the pattern was displayed on the mLED matrix for 0.5 s, followed by another delay of 3 s to eliminate residual effects of the light pattern. The mLED matrix output remained active until the reentry was terminated. Human intervention was thus required only to initiate the reentrant wave.

The experimental protocol for 6‐well format hiAM monolayers was similar to that for the 12‐well format, except for the use of a larger setup and the induction of multiple reentrant waves. A large FOV mapping setup and a larger (160 × 160) mLED matrix were employed to cover the entire monolayer. Multiple reentrant waves were induced using a preprogrammed multi‐S1S2 protocol consisting of 5 sequential S1S2 sequences separated by 100 ms intervals. After confirming successful induction of multiple reentrant waves, the system would automatically terminate individual rotors sequentially based on their significance, with a 3 s delay between each termination.

For multi‐well high‐throughput experiments, reentrant waves were induced simultaneously in four wells using a parallel S1S2 protocol. If induction failed in a particular well, a local S1S2 protocol was applied to establish reentrant waves in that well. To localize reentrant waves across the four wells, the output from the machine learning algorithm was divided into four zones using the software's multi‐well function (See Methods 4.3.1). The mLED output for the bottom‐left well was deliberately turned off as a control, while the outputs for the other wells were automatically turned on 3 s after the induction protocol. The “active” wells were simultaneously illuminated to terminate all reentrant waves at the same time.

### 4.2 Technical Methods

#### 4.2.1 Fabrication of a Flexible mLED Matrix

The mLED matrix was assembled on a flexible substrate made of polyimide (PCBWay, Hangzhou, China), designed in our laboratory using Altium Designer (Altium, Chatswood, Australia). During the assembly process, a layer of flux (SK 10, KONTAKT CHEMIE, Zele, Belgium) was first spray‐coated onto the substrate. The mLEDs (BFB0F11C, 225 × 125 µm^2^, Aucksun, Shenzhen, China) were then placed onto the designated solder pads using pick‐and‐place technology. Next, the mLEDs were secured using a reflow process and coated with optical glue (NOA‐61, Norland Products, Jamesburg, NJ) to protect them from dust and moisture. The fabrication of the substrate and assembly of the mLEDs were carried out in close collaboration with Beijing Charmwin Light Medical Technology Co., Ltd. (Beijing, China). Due to the limited scanning speed of the LED matrix driver, the large mLED matrix was separated into top and bottom zones to reduce the scanning number of each zone. We aimed to keep the refresh rate above a critical threshold to prevent uncontrolled tissue activation [69]. The small matrix operated at 333 Hz, while the large matrix reached 250 Hz. In neither configuration was unwanted activity triggered during the experiments. These frequencies represent the upper performance limit of the current driver hardware. Scaling to larger matrix designs will therefore require more advanced driver designs to sustain sufficiently high refresh rates.

#### 4.2.2 LED Matrix Driver Design

The LED matrix driver consists of three major components (Figure S9a): (1) the microcontroller unit (MCU, RP2040; Raspberry Pi Foundation, Cambridge, United Kingdom), (2) the row driver, and (3) the column driver. Row‐column scanning was used to display dynamic patterns on the mLED matrix. The entire LED matrix driver was coated with a waterproof layer (NANOCOAT200‐10‐500ML; Mouser Electronics, Munich, Germany).

##### 4.2.2.1 Row Driver

In the scanning operation, the LEDs are turned on row by row at high speed to display a specific light pattern. This is achieved using shift registers, which store the on/off status of each row, and a p‐channel metal‐oxide–semiconductor (PMOS) array that acts as a switch. In this configuration, the anodes of all LEDs in the same row are connected in parallel to the LED power supply through a PMOS (DMP2065UFDB‐7; Diodes, Plano, TX). The output of the shift register (74HC164BQ115; Nexperia, Nijmegen, the Netherlands) controls the gate of the PMOS. The shift registers are controlled by the MCU via two signal lines: a clock and a data line.

##### 4.2.2.2 Column Driver

During the scanning process, when a given row is activated, specific LEDs within that row are turned on according to the desired light pattern. To achieve individual column control, 16‐channel programmable constant‐current LED drivers (CAT4016, OnSemi, Scottsdale, AZ) are used. Each driver contains a 16‐bit built‐in shift register capable of storing the on/off state of 16 LEDs. Unlike the 74HC165 shift register, the CAT4016 includes a latch between the register and the output, ensuring that the LED outputs remain unchanged until a latch signal is received. Global dimming of the LED matrix is accomplished by applying a 1 MHz pulse‐width modulation signal to the enable pin of the current source.

##### 4.2.2.3 Scalability

The driver design is scalable by adjusting the number of row shift registers and column constant‐current sources. The printed circuit board design shown in Figure S9b corresponds to the 48 × 32 mLED matrix driver, while Figure S9c shows the driver for the 160 × 160 mLED matrix.

#### 4.2.3 Illumination Uniformity

Because our mLED matrices consist of emitters rather than a continuous light source, they can produce nonuniform illumination on the receiving plane. Illumination uniformity depends on the pitch between light sources (D) and the distance to the receiving plane (H), which can be captured by their ratio (σ = D/H). To investigate how uniformity varies with σ, numerical simulations were performed.

Each mLED was modeled as a Lambertian emitter, which is a reasonable approximation given its small size. Figure S3a,b shows simulation results for a 32‐LED array with σ = 10 and σ = 1, respectively. As expected, reducing σ from 10 to 1 substantially improved illumination uniformity.

Illumination uniformity was quantified as the ratio of the minimum to maximum light intensity sampled in the central region of the LED array. An ideal, perfectly uniform illumination corresponds to a uniformity value of 1. Simulations across a range of σ values yielded the relationship shown in Figure S3c. The results indicate that achieving at least 80 % uniformity requires σ< 3.

In our experimental setup, the distance from the mLED surface to the cultured monolayer in the well plate is approximately 3 mm, accounting for both the well plate height and the isolation layer above the mLEDs. To achieve the desired illumination uniformity of 80%, the center‐to‐center spacing between adjacent mLEDs must therefore be below 1 mm. Both of our matrix designs meet this requirement, with pitches of 0.6 and 0.9 mm.

#### 4.2.4 Thermal Characteristics

The temperature rise of the mLED matrix was assessed using a thermal infrared camera (VarioCAM, InfraTec). Before measurement, the matrix was preheated to 37°C to approximate physiological conditions. The matrix was then operated at maximum power with all mLEDs activated for 5 min to reach a thermally stable state. The resulting thermal image is shown in Figure S4a. The apparent temperature nonuniformity is attributed to the uneven surface of the optically transparent (not infrared transparent) coating covering the mLEDs.

To evaluate transient heating during optical stimulation, we applied a protocol consisting of five light pulses (500 ms on, 500 ms off) with the entire matrix activated. Figure S4b displays the average temperature change within the region of interest (indicated by the thin white rectangle in Figure S4a) under different pulse‐width modulation (PWM) settings. The corresponding peak temperature rise at the hottest location during each pulse is shown in Figure S4c.

#### 4.2.5 Communication Protocol

Communication between the LED matrix driver and the personal computer was established via Serial‐over‐USB. To transmit custom patterns, the state of each LED was represented as 0 (off) or 1 (on), with every eight LEDs encoded into a single byte. The total number of bytes required was equal to the total number of pixels divided by eight. For an 80 × 160 pixel frame (large mLED matrix), a measured transmission time per frame was 15 ms, including the coding and decoding time.

### 4.3 Computational Methods

#### 4.3.1 Real‐Time OVM System

To enable real‐time optical mapping, we developed custom software in Python 3.12 (Python Software Foundation, Beaverton, OR). To accelerate processing, a multi‐threading architecture was implemented to distribute computational tasks across multiple CPU cores (Figure S13a).

The first thread handled image processing. Frames were acquired directly from the MiCAM05 optical mapping system (SciMedia, Costa Mesa, CA) into our software via an application programming interface provided by BrainVision (Morrisville, NC). Data packs of five raw frames (100 × 100 × 5) were collected at 8 ms intervals. The last frame of a pack served as background for the subsequent pack. This background was subtracted so that the remaining image data contained only dynamic changes relative to the prior frame, realizing a high‐pass filter effect. Next, a Gaussian low‐pass filter with a 3 × 3 kernel was applied to the frames to reduce noise. Finally, the frames were cropped to 86 × 86 × 5 to match the input size of the machine learning algorithm for spiral core detection. The machine learning algorithm returned the detection result in an 86 × 86 array with values ranging from 0 to 1, indicating the likelihood of a spiral core being present at each pixel. These steps took 61.180 ± 6.580 ms across 23 loops for image acquisition, 4.650 ± 4.415 ms for image preparation, and 12.338 ± 7.639 ms for the ML processing stage (Figure S13c).

The second thread processed the machine learning algorithm output from the image‐processing thread to identify spiral core locations. To do so, the coordinates of the maximum values from 10 consecutive frames were accumulated into a histogram. If the average of these maximum values exceeded 0.7 (70% likelihood of a spiral core being present in this location), the program returned the maximum value coordinate in the histogram as the spiral core location. A light pattern in the form of a line connecting the spiral core to the nearest border was automatically generated. A user‐friendly interface (UI) (Figure S16) allowed the mLED matrix output to be enabled or disabled, while simultaneously displaying the raw image, the machine learning detection result, and the generated light patterns in real time. The UI also allowed easy modification of program parameters such as light pattern delay (3 s by default, see Methods 4.1.3) and duration (0.5 s by default, see Methods 4.1.3).

The third thread handled the communication between the program and the LED matrix driver. After a pattern was generated in the second thread (5.380 ± 4.300 ms, Figure S13c), it was sent to the mLED matrix (59.033 ± 18.112 ms, Figure S13c) while the second thread simultaneously rendered the UI. As a result, the actual light pattern displayed on the mLED matrix was synchronized with the display on the UI.

Communication with the monolayer constitutes the most time‐consuming step (Figure S13c) and was therefore distributed across two separate threads. The overall loop time (79.943 ± 13.416 ms) was determined by the slowest individual thread. To further minimize loop time, the program was tested on three different personal computers, showing that a fast CPU could reduce it to as little as 100 ms (Figure S13b).

To enable high‐throughput multi‐well reentry termination, the output of the machine learning algorithm was divided into 4 quadrants corresponding to the four well locations (Figure S16 left). The coordinates of maximum‐value positions in each quadrant were identified and displayed on the UI (Figure S16 middle). When the LED matrix output was enabled, a light pattern consisting of lines connecting the cores of reentrant waves with a score above 0.7 to the nearest border was created (Figure S16 right). The LED matrix output for each well could be individually controlled via the UI. In the reentry termination experiment, the output for the bottom‐left well (well 3) was deliberately disabled as a control.

#### 4.3.2 Synthetic Data Generation for Training the CNN

Electrical waves were simulated using a neonatal rat ventricular myocyte model [42] (Figure S10). This single‐cell model was assigned to every voxel of a 2D grid representing a cardiac monolayer. This grid contained 256 × 256 points, each measuring 60 × 60 µm^2^, yielding a total surface of size 15.36 × 15.36 mm^2^. In the center of each virtual monolayer, a circle with a diameter of 15 mm (mimicking the area of a well in a 24‐well culture plate) was defined as conducting, while the remaining parts of the grid were non‐conducting. Neumann boundary conditions (0‐weights between grid points) were applied at the circle's border.

For each virtual monolayer, all ionic currents were globally varied from 83% to 120% of their standard amplitudes to generate sample‐to‐sample variability. Additionally, for each cardiomyocyte equation (corresponding to each node of the simulation grid), these currents were independently varied from 50% to200% of the global value to impose realistic cell‐to‐cell ionic heterogeneity. Tissue conduction heterogeneity was introduced by varying the coupling coefficient between cardiomyocytes from ¼ × to 4 × its standard value.

Due to this heterogeneity, not all samples could sustain spiral waves, and some could not even support wave propagation. Such failing samples accounted for approximately 25% of all simulations and were excluded from further analysis.

Spiral waves were generated using an S1S2 protocol, with the S2 stimulus applied at a random location within the 15 mm circle of conducting cells. Once the spiral was stable, each virtual monolayer was simulated for a total real‐time duration of 1.25 s with a time step of 0.005 ms. Images were recorded every 1200 steps, producing a 208‐frame video with a temporal resolution of 6 ms, equal to experimental optical mapping videos of neonatal rat ventricular myocyte layers.

#### 4.3.3 Spiral Center Detection Network: A CNN

To detect the spiral centers, which are key features in the dynamical behavior of an arrhythmia, a dedicated deep learning algorithm was developed. A CNN was used to account for the translational symmetry (in both space and time) of the video recordings. We specifically chose a CNN because this property enables reliable detection of spiral tips regardless of their position in the field of view. CNNs naturally learn hierarchical representations: shallow layers capture fine‐grained spatial details, while deeper layers integrate broader contextual information. This hierarchy is essential for moving beyond coarse classification and achieving precise localization. The combination of local detail and global context also supports robust detection of spiral centers in moderately noisy data [48]. A moderately sized CNN was chosen to balance accuracy and efficiency. Such a model provides sufficient representational capacity for this task without unnecessary over‐parameterization. In contrast, substantially deeper or more modern architectures would require much larger training datasets and impose additional computational overhead, without offering meaningful performance gains for this application.

Our CNN network consists of six layers, depicted in Figure S11a, with each layer having dimensions C × T × W × H (Channels × Time × Width × Height). In these visualizations, width and height are represented as depth and height, while the thickness of each sub‐layer indicates the number of channels (C). The time dimension is illustrated by duplicating sub‐layers, thereby forming the total layer. For example, the input layer of this CNN consists of 5 consecutive greyscale video frames (one channel each), each with a width and depth of 86 pixels. This is visualized as a 1 × 86 × 86 sub‐layer duplicated five times, forming a 1 × 5 × 86 × 86 input layer.

Spatial gradients were computed for each frame in this input layer to encode advanced spatial information [70]. This was followed by a dimension‐reducing convolution layer (3 × 3 × 3 kernel) and a max‐pooling layer (3 × 3 × 3 kernel, stride 1 × 2 × 2) layer, resulting in a layer of dimension 16 × 1 × 39 × 39. The data then passed through another convolution layer (1 × 3 × 3 kernel) and a max‐pooling layer (1 × 3 × 3 kernel, stride 1 × 2 × 2), followed by decoding via two 2D deconvolution operations. The final output was interpolated to match the input dimensions (86 × 86 pixels), yielding an image in which each pixel represents the probability of a spiral center.

The network was trained on a dataset of 7776 spiral patterns, split 80/20 into training and validation sets. The loss function consisted of an L_1_‐norm with an additional penalty for near‐zero solutions, which prevents the model from collapsing into a trivial solution (i.e., always predicting “no spiral”). The loss function was optimized using the Adam [71] optimizer for 100 epochs.

## Author Contributions

Conceptualization: Shanliang Deng, Niels Harlaar, Nina N. Kudryashova, Alexander V. Panfilov, Tim De Coster, Daniël A. Pijnappels. Methodology: Shanliang Deng, Niels Harlaar, Juan Zhang, Sven O. Dekker, Nina N. Kudryashova, Huiling Zhou, Cindy I. Bart, TianYi Jin, Tim De Coster. Software: Shanliang Deng, Nina N. Kudryashova, Georgy Derevyanko, Tim De Coster. Validation: Shanliang Deng, Niels Harlaar, Tim De Coster. Formal analysis: Shanliang Deng, Tim De Coster. Investigation: Shanliang Deng, Niels Harlaar, TianYi Jin, Tim De Coster. Resources: Antoine A. F. de Vries, GuoQi Zhang, Daniël A. Pijnappels. Data curation: Daniël A. Pijnappels. Writing – original draft: Shanliang Deng, Tim De Coster. Writing – review & editing: Niels Harlaar, Juan Zhang, Sven O. Dekker, Nina N. Kudryashova, Huiling Zhou, Cindy I. Bart, TianYi Jin, Georgy Derevyanko, Willem van Driel, Alexander V. Panfilov, René H. Poelma, Antoine A. F. de Vries, GuoQi Zhang, Daniël A. Pijnappels. Visualization: Shanliang Deng, Tim De Coster. Supervision: GuoQi Zhang, Tim De Coster, Daniël A. Pijnappels. Project administration: Daniël A. Pijnappels. Funding acquisition: Daniël A. Pijnappels.

## Funding

This work was supported by the European Research Council (ERC consolidator grant 101044831 to DAP).

## Conflicts of Interest

The authors declare no conflicts of interest.

## Supporting information




**Supporting File 1**: advs74173‐sup‐0001‐SuppMat.docx.

**Supporting File 2**: advs74173‐sup‐0002‐FigureS1‐S16.zip.

**Supporting File 3**: advs74173‐sup‐0003‐VideoS1‐S3.zip.

## Data Availability

The data that support the findings of this study are available from the corresponding author upon reasonable request.
