# Supplementary material for: Smart Optogenetics for Real‐Time Automated Control of Cardiac Electrical Activity
Source: Adv Sci (Weinh). 2026 Feb 13;13(20):e22759. doi: 10.1002/advs.202522759 (PMC13067851; doi:10.1002/advs.202522759)
Supplement: Supplementary file 1 — Supporting File 1: advs74173‐sup‐0001‐SuppMat.docx. [file ADVS-13-e22759-s002.docx]

Supporting Information

Smart optogenetics for real-time automated control of cardiac electrical activity

Shanliang Deng, Niels Harlaar, Juan Zhang, Sven O. Dekker, Nina N. Kudryashova, Huiling Zhou, Cindy I. Bart, TianYi Jin, Georgy Derevyanko, Willem van Driel, Alexander V. Panfilov, René H. Poelma, Antoine A.F. de Vries, GuoQi Zhang, Tim De Coster^†^, Daniël A. Pijnappels^†,^*

^†^ These authors contributed equally

* Contact: d.a.pijnappels@lumc.nl

**1 Supporting Video Legends**

**Video SV1.** Real-time closed-loop mapping of programmed initiation and automatic termination of a single reentrant circuit in a single cardiac monolayer. Video recording used to produce Figure 4: Left panel: Raw images at different stages of reentry wave progression. Middle panel: ML-generated maps showing the predicted spiral core locations. Right panel: Histogram arrays tracking the most probable core positions over the previous 10 frames, with the current core marked as a red dot in the raw images.

**Video SV2.** Real-time closed-loop mapping of programmed initiation and automatic termination of multiple reentrant circuits in a single cardiac monolayer. Video recording used to produce the middle row of Figure 6: Left panel: Raw images at different stages of reentry wave progression. Middle panel: ML-generated maps showing the predicted spiral core locations. Right panel: Histogram arrays tracking the most probable core positions over the previous 10 frames, with the current core marked as a red dot in the raw images.

**Video SV3.** Real-time closed-loop mapping of programmed initiation and automatic termination of a single reentrant circuit in multiple cardiac monolayers. Video recording used to produce the bottom row of Figure 6: Left panel: Raw images at different stages of reentry wave progression. Middle panel: ML-generated maps showing the predicted spiral core locations. Right panel: Histogram arrays tracking the most probable core positions over the previous 10 frames, with the current core marked as a red dot in the raw images.

**2 Supporting Figures**


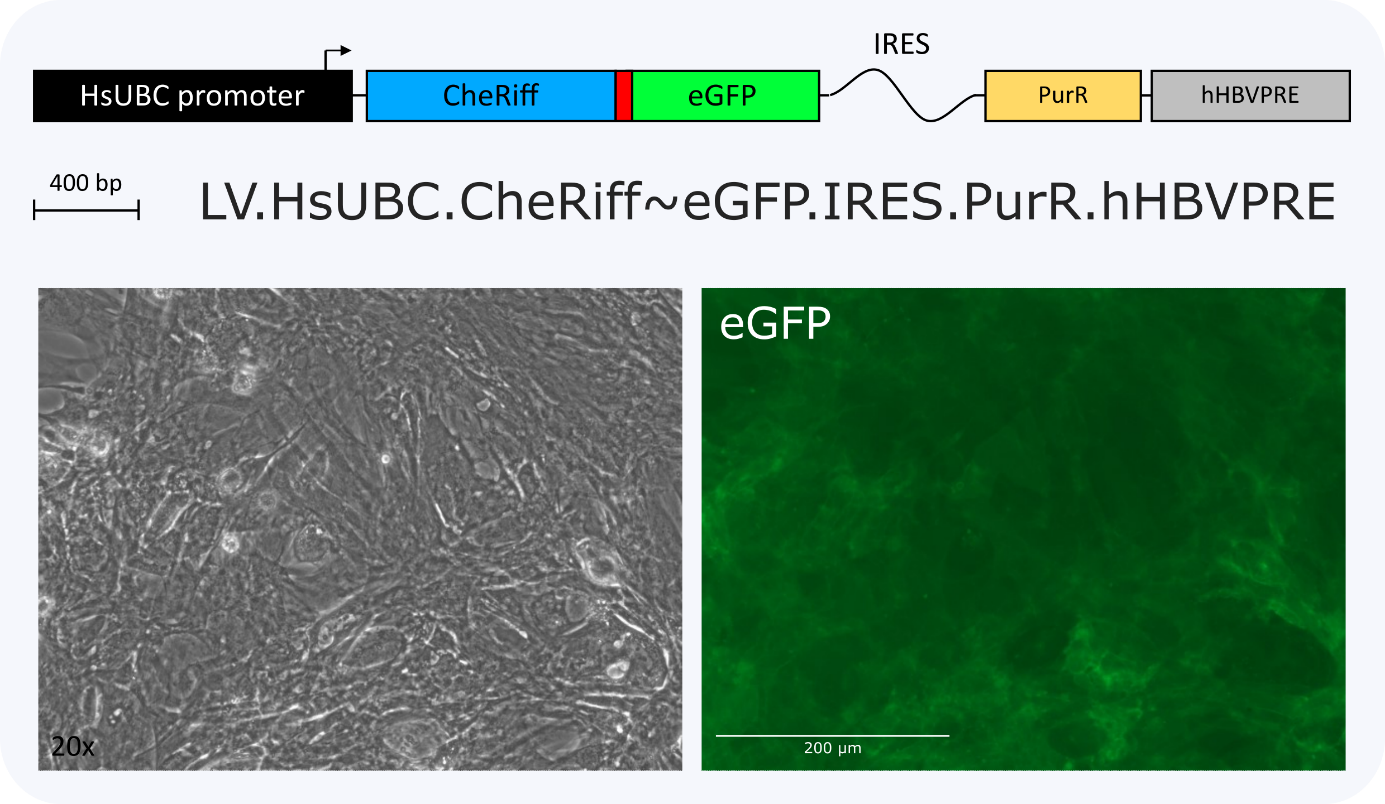


**Figure S1.** CheRiff-hiAMs. Top: Schematic of the plasmid construct used to generate the self-inactivating lentiviral vector LV.HsUBC.CheRiff~eGFP.IRES.PurR.hHBVPRE for optogenetic modification of hiAMs. Bottom: Bright-field image (left) and corresponding fluorescence image of CheRiff~eGFP-expressing cells (right).

**
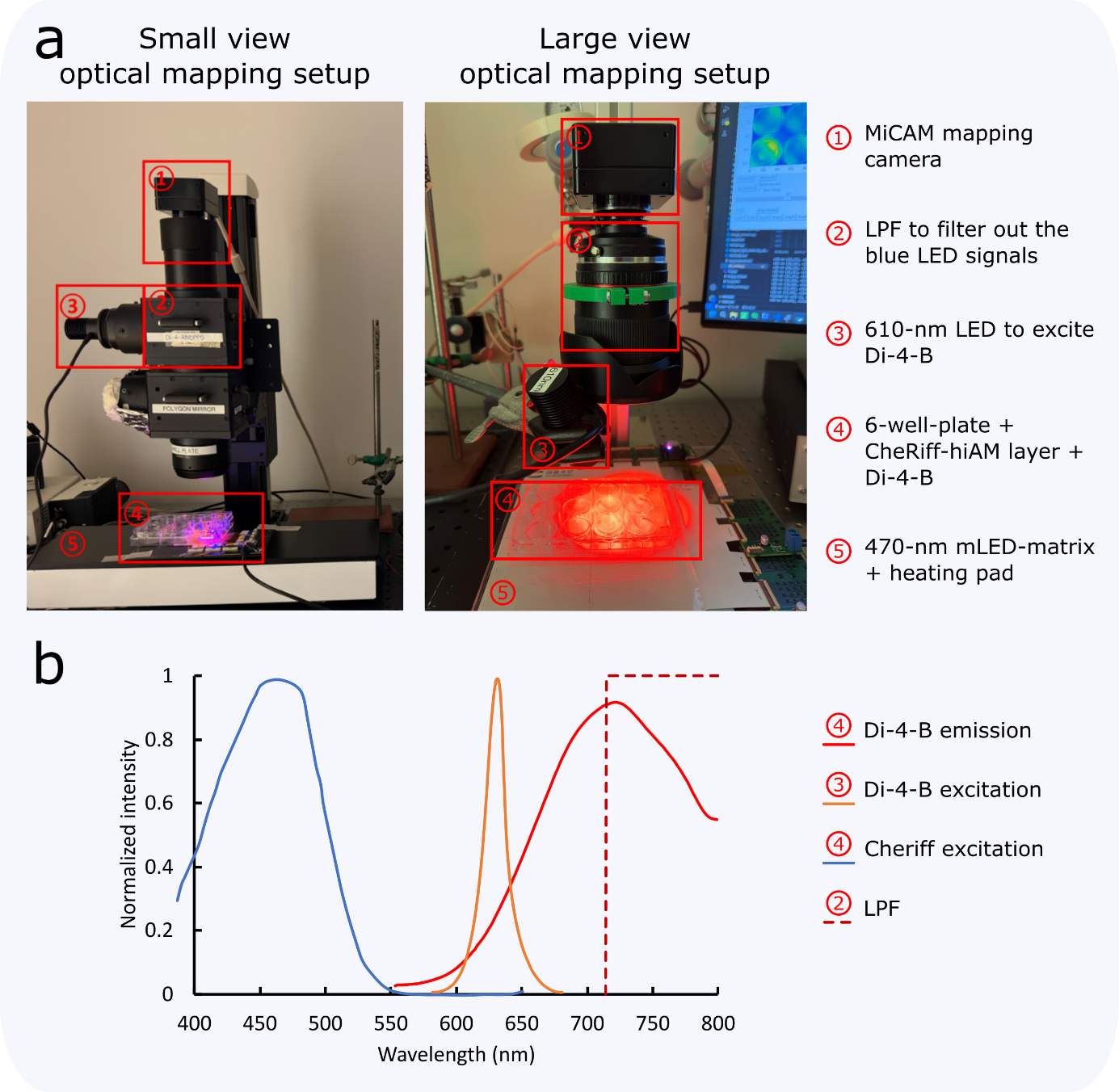
Figure S2.** Overview of the separate components in the optical mapping and manipulation setups. (a) Images of the standard OVM system (left) and the large field-of-view OVM system (right), with key components labeled. (b) Normalized spectral distribution of the system’s optical elements. Red: Di-4-ANBDQBS (Di-4-B) emission; orange: LXZ1-PH01 excitation: blue; CheRiff activation; deep red: long-pass filter (LPF) transmission range.


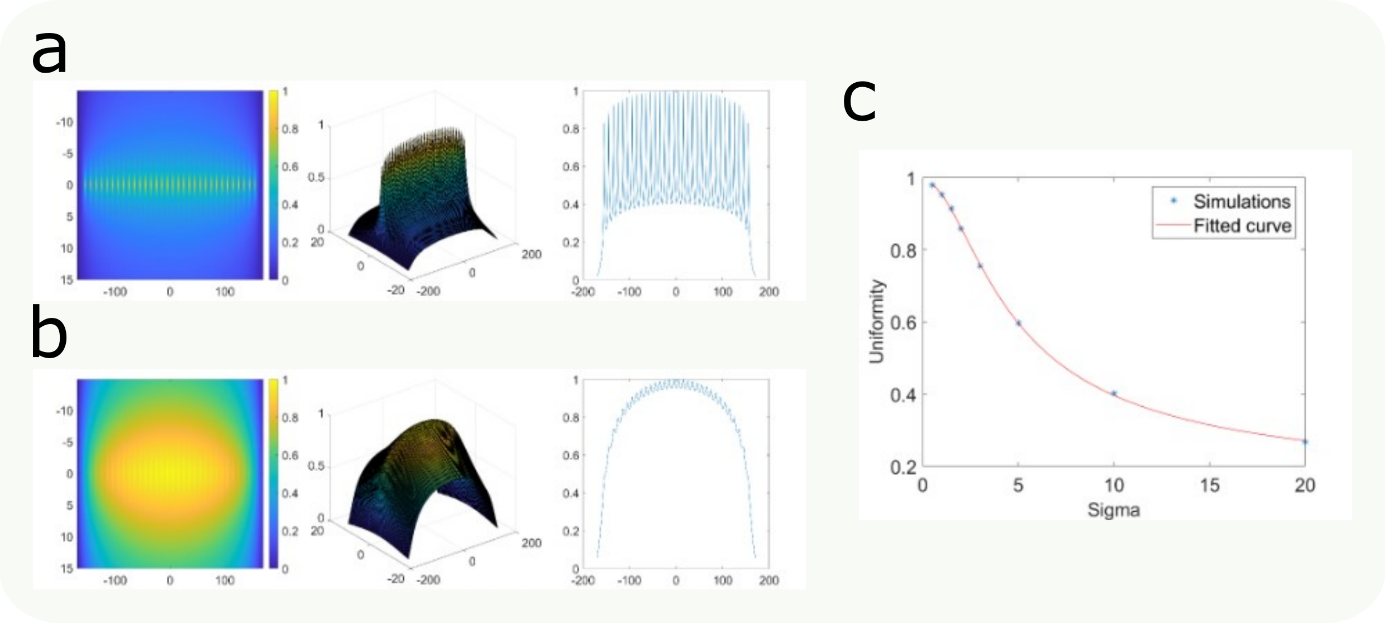


**Figure S3.** Illumination uniformity of the mLED matrix. (a-b) Left: Normalized surface light intensity. Middle: Three-dimensional spatial light profile. Right: Normalized two-dimensional spatial light distribution of an mLED array modeled using a Lambertian emission profile. Results are shown for σ = 10 (a) and σ = 1 (b). (c) LED array illumination uniformity as a function of σ. Blue dots represent simulated values for different σ, and the red curve shows the fitted curve to the simulation data.


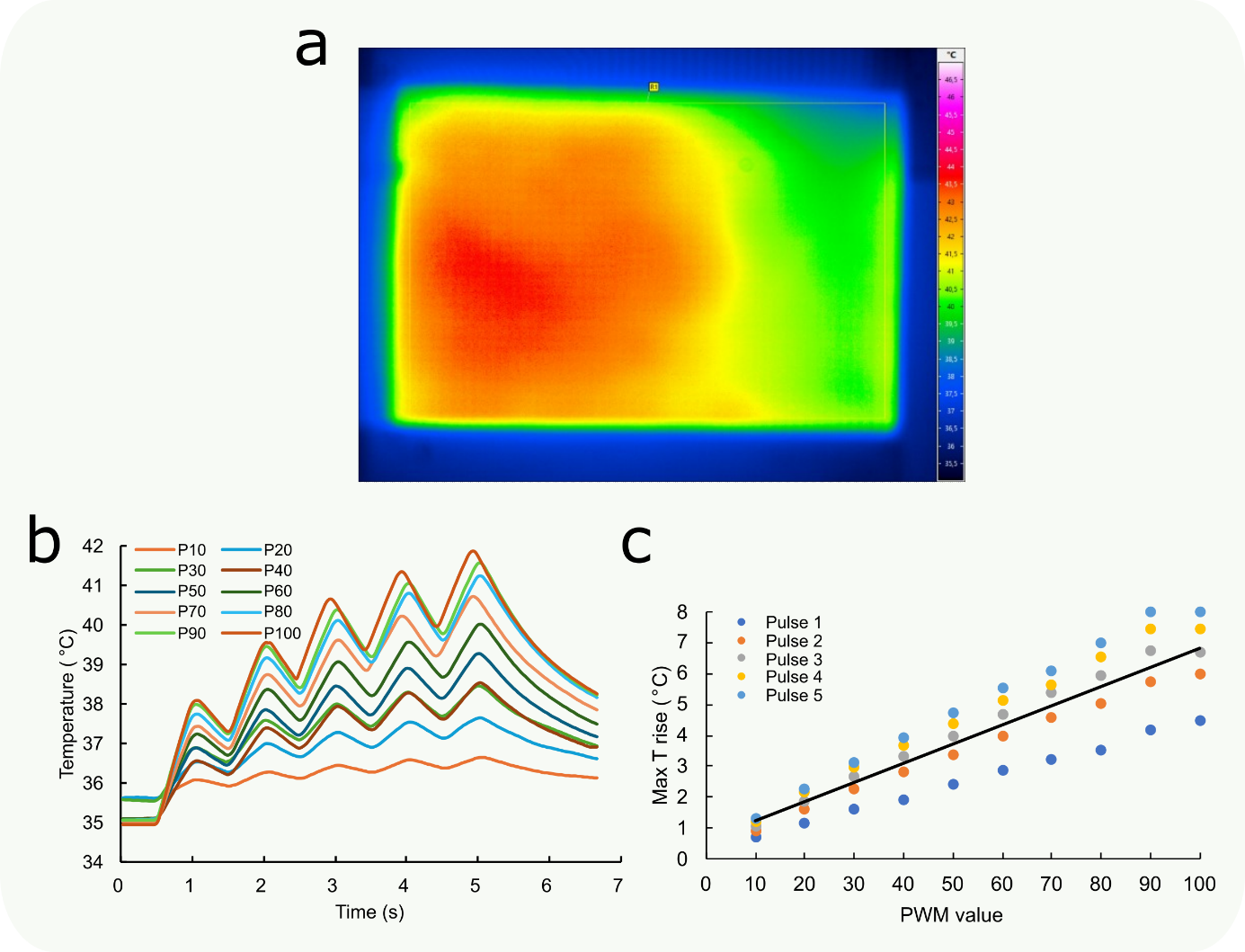
**Figure S4.** Thermal characteristics of double the small mLED matrix (48 × 64). (a) Thermal image of the mLED matrix operating at maximum power under steady-state conditions with all LEDs on. The white rectangular outline indicates the region used to compute the average temperature. (b) Average temperature change of the LED matrix during five light pulses (500ms on, 500ms off) at different pulse-width modulation (PWM) values, with all LEDs activated. (c) Maximum temperature increase of the LED matrix for each of the five pulses at different PWM values.


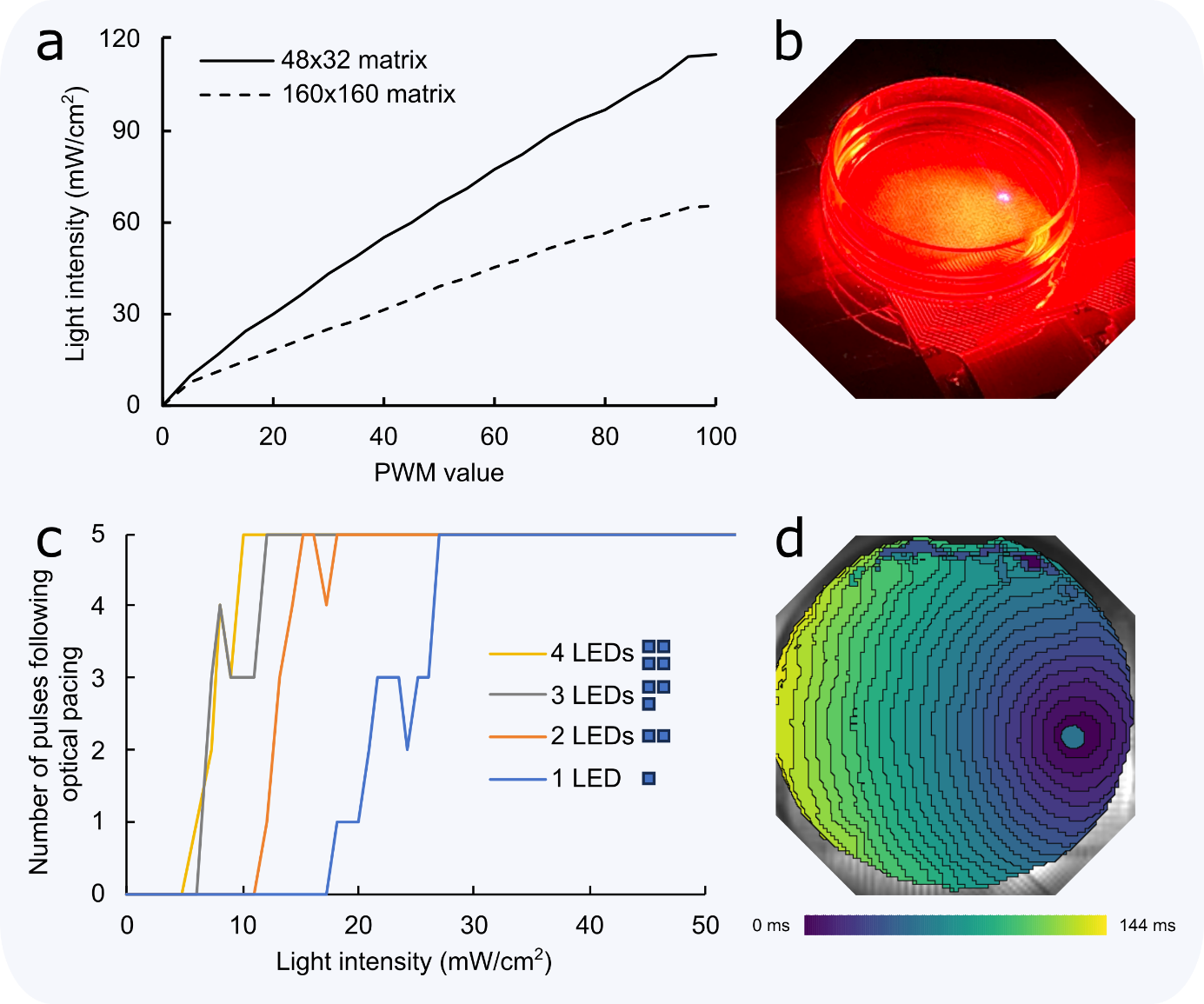


**Figure S5.** Optical pacing parameters of the mLED matrix. (a) Light intensity (mW cm^-2^) as a function of pulse-width modulation (PWM) value for the large (dashed line) and small (solid line) mLED matrices. (b) Optical pacing of a CheRiff-hiAM monolayer using three LEDs (450 nm) from each mLED matrix. (c) Number of excitation waves (pulses) elicited as a function of light intensity (mW cm^-2^) and the number of active mLEDs. (d) Activation map of the electrical wave generated in (a).


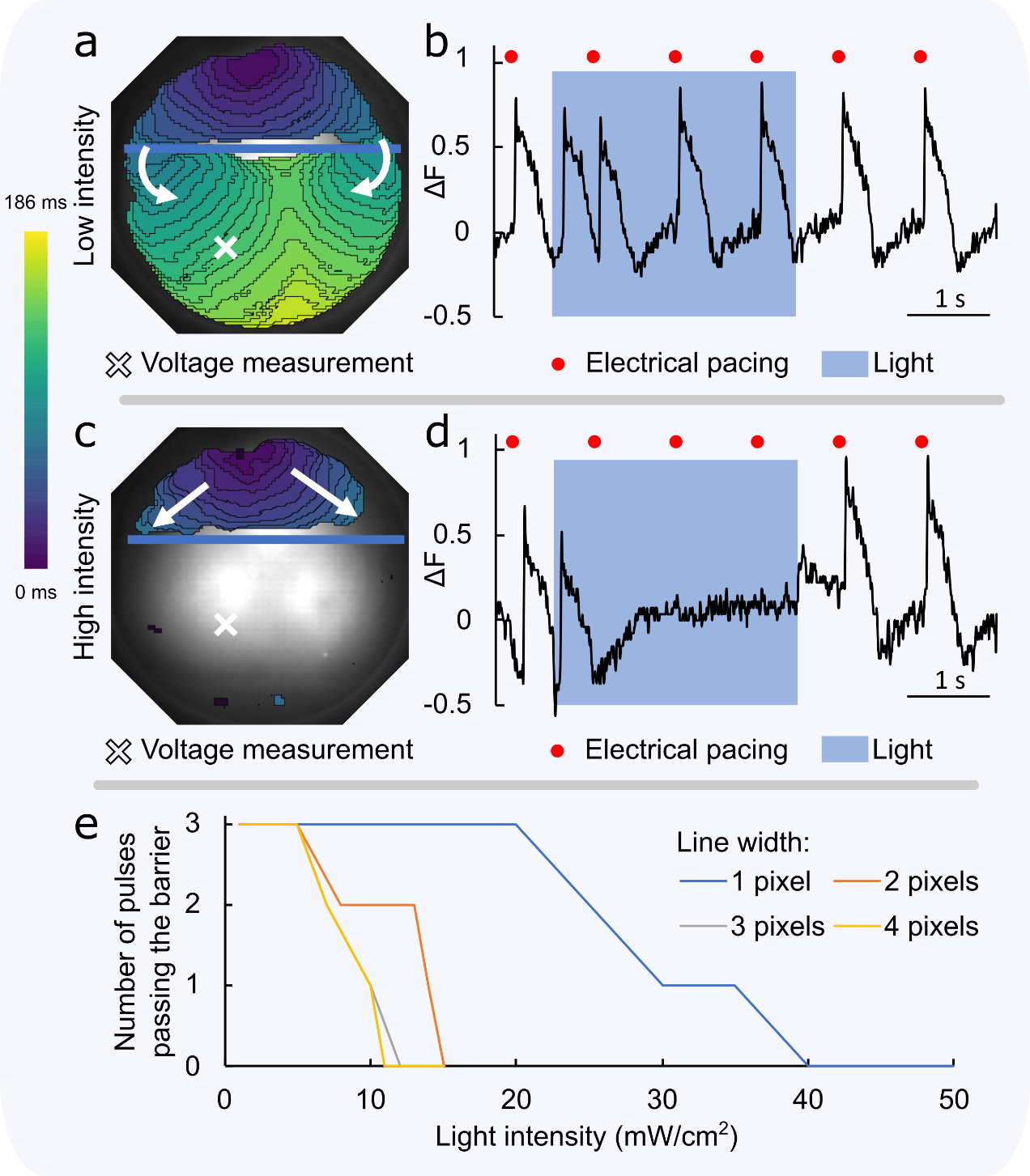


**Figure S6.** Demonstration of conduction block using the mLED matrix. (a) Activation map of a progressing wave under conduction block created by a single line from the mLED matrix with a light intensity of 10 mW cm^-2^. The action potential measurement site is indicated by a cross. (b) Fluorescence change (ΔF) over time at low light intensity, showing that three paced excitation waves still pass through the blocking region (the first action potential in the blue region originates from switching on the blue light). (c) Activation map of a progressing wave under conduction block created by a single line from the mLED matrix with a light intensity of 40 mW cm^-2^. The action potential measurement site is indicated by a cross. (d) Fluorescence change over time at high light intensity, showing complete blockage of wave propagation. (e) Conduction block efficiency (number of pulses out of three passing the blocking line) for different line thicknesses and light intensities.


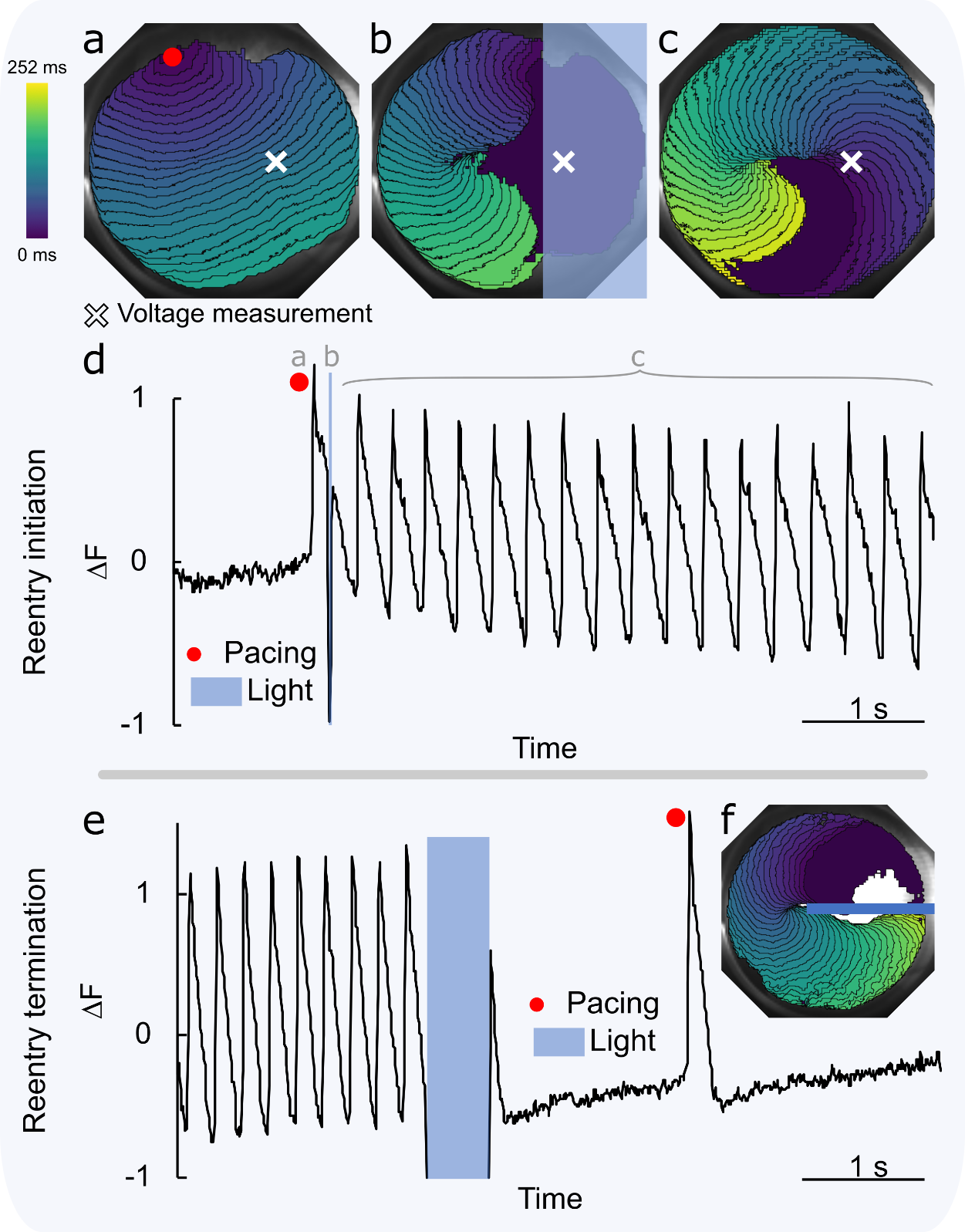


**Figure S7.** Optogenetic manipulation of reentry wave initiation and termination using the mLED matrix. (a-c) Activation maps of a CheRiff-hiAM monolayer after the S1 pulse (red dot) (a), S2 pulse (blue rectangle) (b) and established reentry (c), illustrating the S1S2 protocol to induce a reentrant wave. (d) Fluorescence change (ΔF) over time showing the induction of a reentrant wave, in which the S1 pulse is marked by the red dot (a) and the S2 pulse by the blue box (b). (e) Fluorescence change during reentrant wave termination using light-induced conduction block (blue box) followed by electrical sinus rhythm pacing (red dot). (f) Activation map of successful reentry termination, with the blue area indicating the applied light pattern.


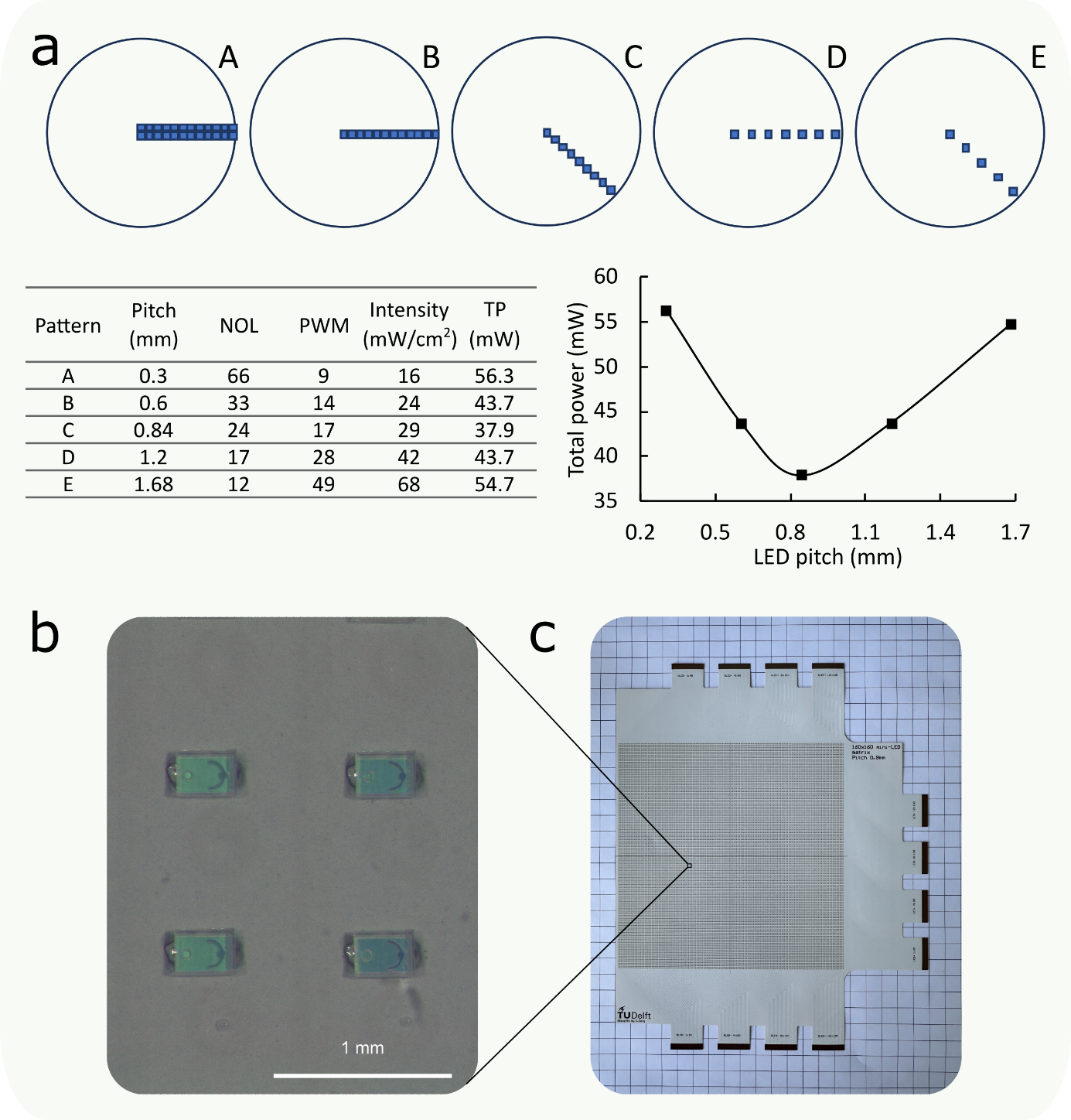


**Figure S8.** Power efficiency using patterned illumination for reentrant wave termination. (a) Top: Five line patterns (1-5) with varying LED pixel pitches (0.3 mm to 1.68 mm). Left: Line properties required to terminate a reentrant wave: number of LEDs per line (NOL), pulse-width modulation (PWM), light intensity, and total power (TP). Right: TP required for wave termination as a function of LED pixel pitch. (b) Microscopic image of a small portion of the large-size mLED matrix. (c) Large-size mLED matrix placed on 1-cm grid paper.


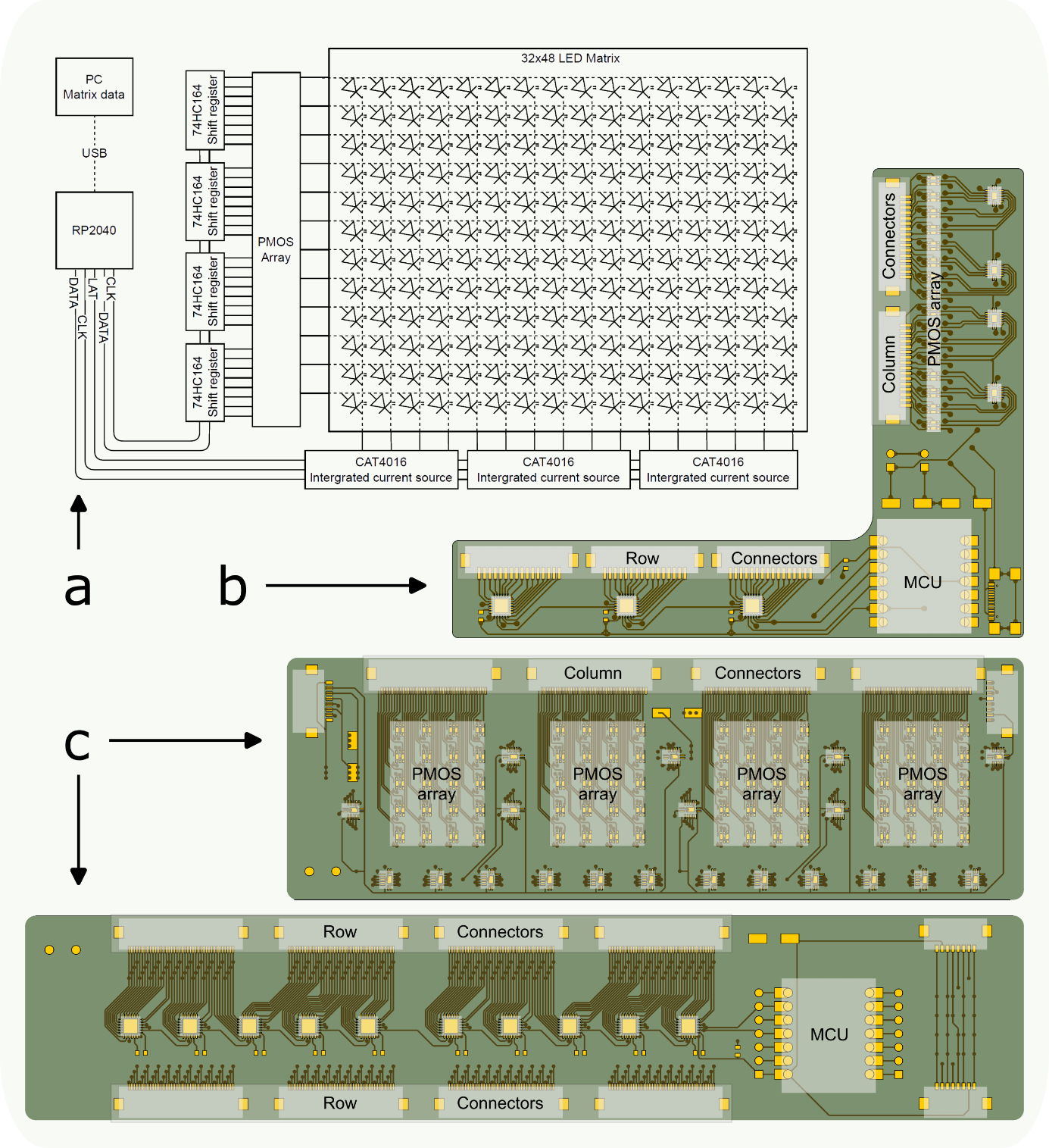


**Figure S9.** Hardware design of the mLED matrix drivers. (a) Circuit schematic of the small-size mLED matrix driver, showing all components and connections. (b) Printed circuit board (PCB) layout of the small-size mLED matrix driver, with key components labeled: row and column connectors, microcontroller unit (MCU), and p-channel metal-oxide semiconductor (PMOS) array. (c) PCB layouts of the large-size mLED matrix driver, with the same key components labeled. Top: column driver PCB, Bottom: row driver PCB.


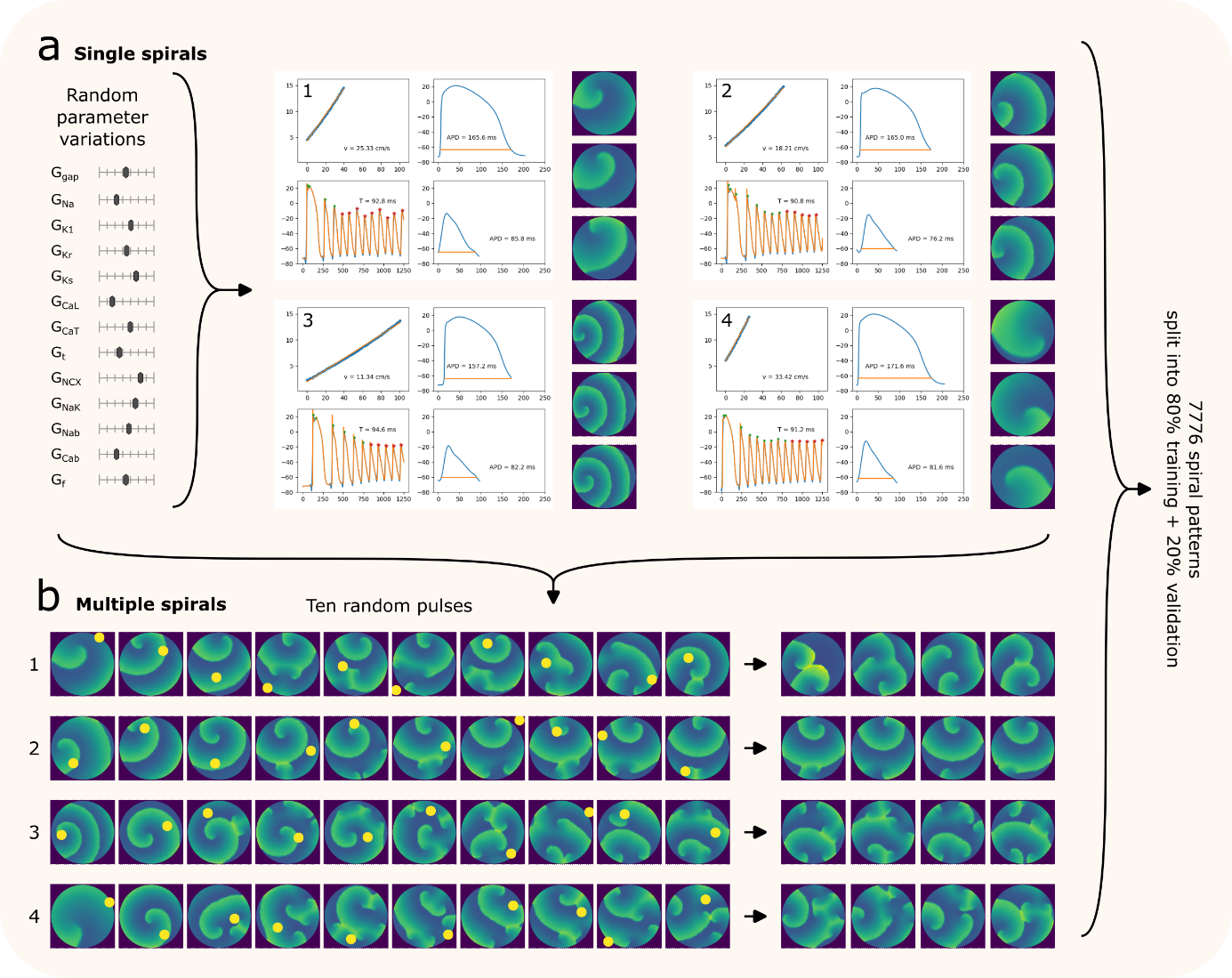


**Figure S10.** *In silico* data generation. (a) Heterogeneous tissue samples were created by varying 13 parameters. In each sample, a spiral wave was induced using the S1S2 protocol. Key characteristics of each monolayer, i.e. action potentials (APs), initial and final AP duration (APD) at 90% repolarization, rotational period (T) and conduction velocity (v), were recorded in “passports” with four examples shown. (b) More complex spiral patterns were generated by applying 10 random pulses applied to the initial set of single spirals, producing 7776 spiral patterns in total, which were split 80/20 for training and validation.

**
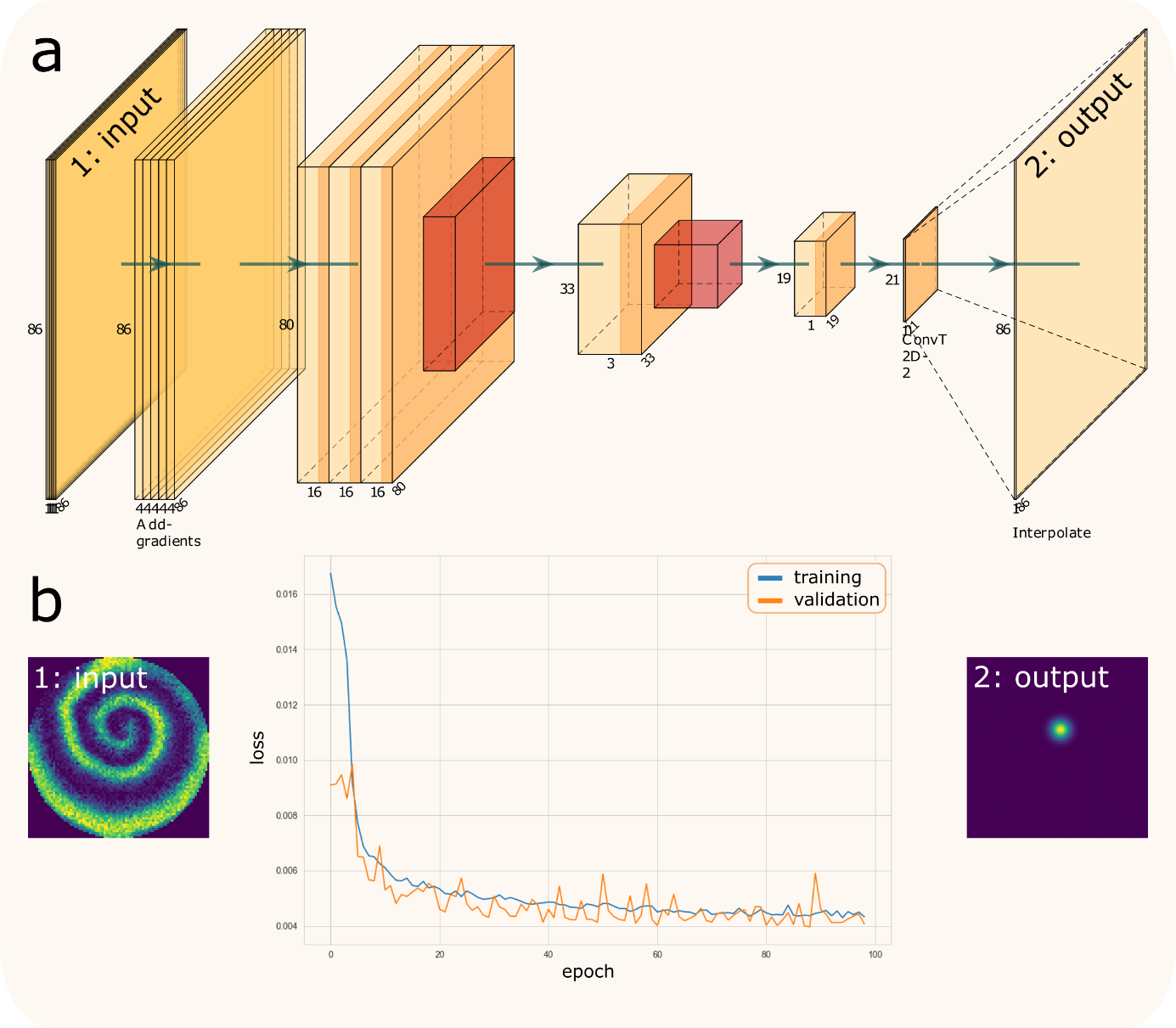
Figure S11.** Software design of the neural network and graphical user interface. (a) Schematic of the neural network architecture used for spiral center detection. The network takes five sequential video frames as input (1×5×86×86) and outputs a single image showing the probability density of spiral centers (1×1×86×86). (b) Training and validation loss for our neural network.


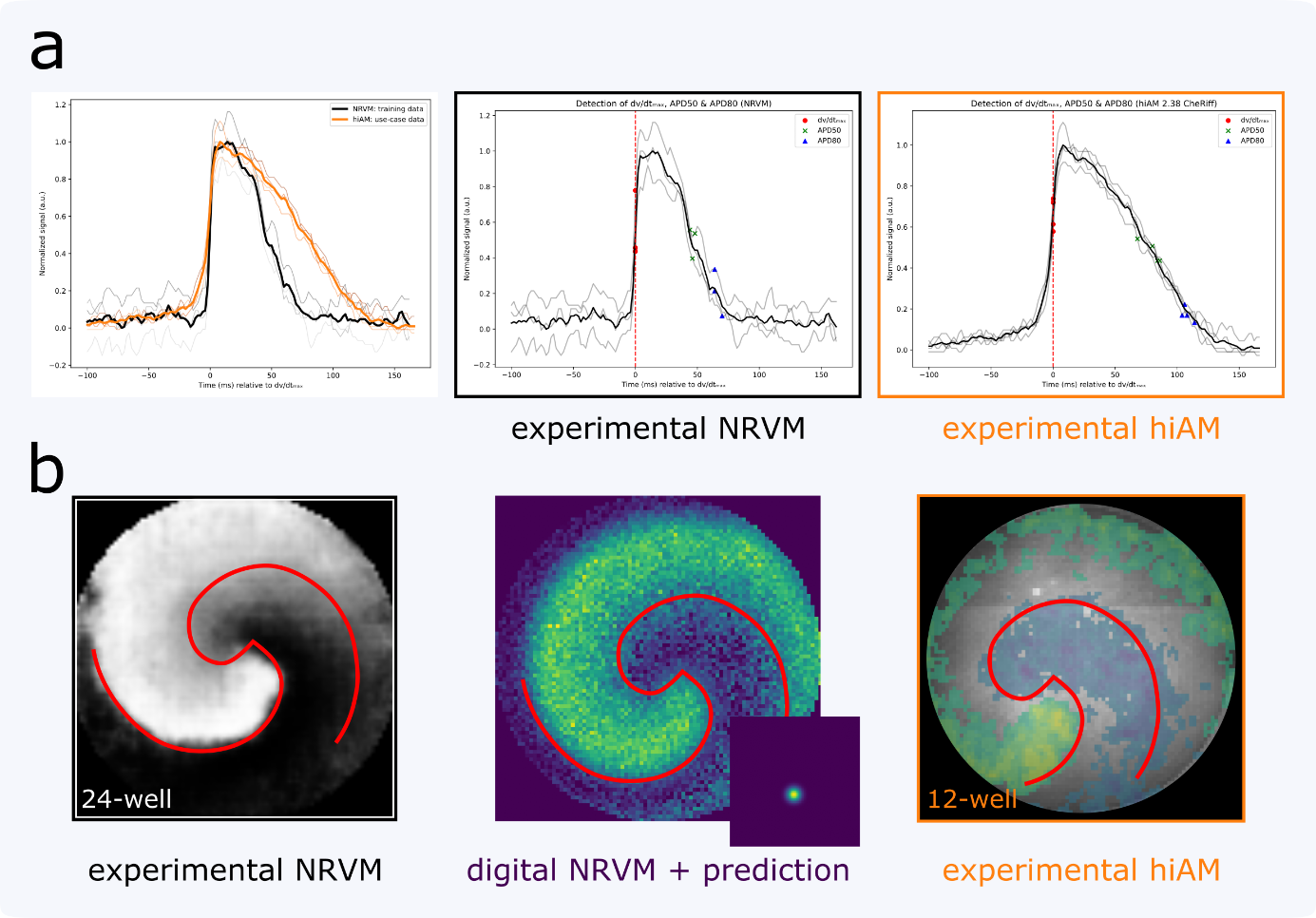


**Figure S12.** Differences and similarities between NRVMs and hiAMS. (a) Experimental action potential morphology for training data (NRVMs) and use-case data (hiAMs). Traces were extracted from optical mapping data recorded under 1-Hz electrical pacing. Action potentials (4 for each cell type) were overlaid and averaged to highlight differences in morphology. To allow direct comparison, action potential amplitudes were normalized. normalized. (b) Spiral morphology in experimental (NRVM and hiAM) and simulated monolayers exhibits the same shape, with the red curve indicating identical curvature. This consistency is independent of cell type and well size, confirming robust CNN performance based solely on visual input.


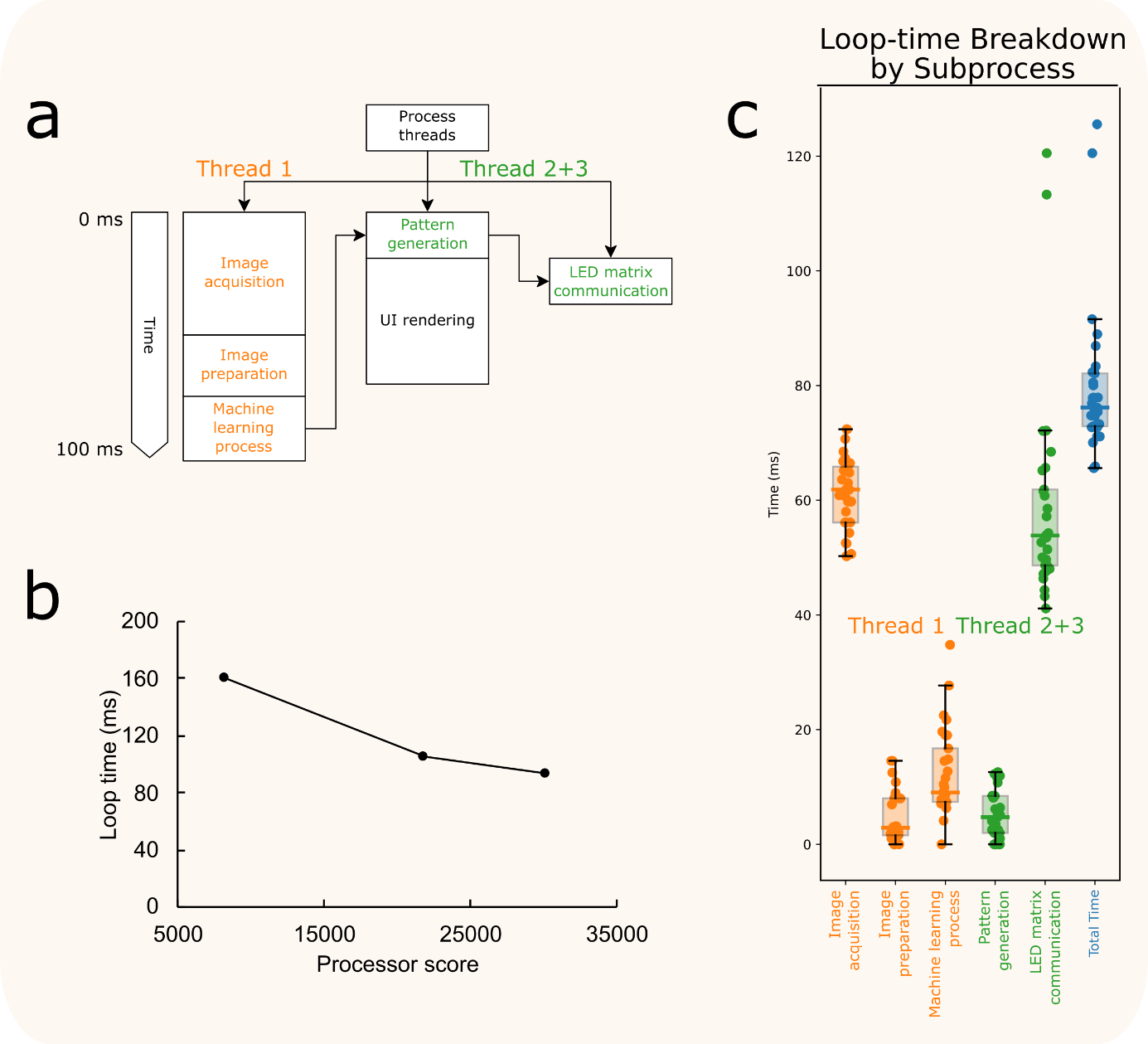


**Figure S13.** Loop-time. (a) Schematic of the processing threads enabling parallel execution of all software components for efficient computation. (b) Software performance as a function of processor score, showing an asymptotic limit near 100 ms. (c) Breakdown of the total loop time by subprocess.


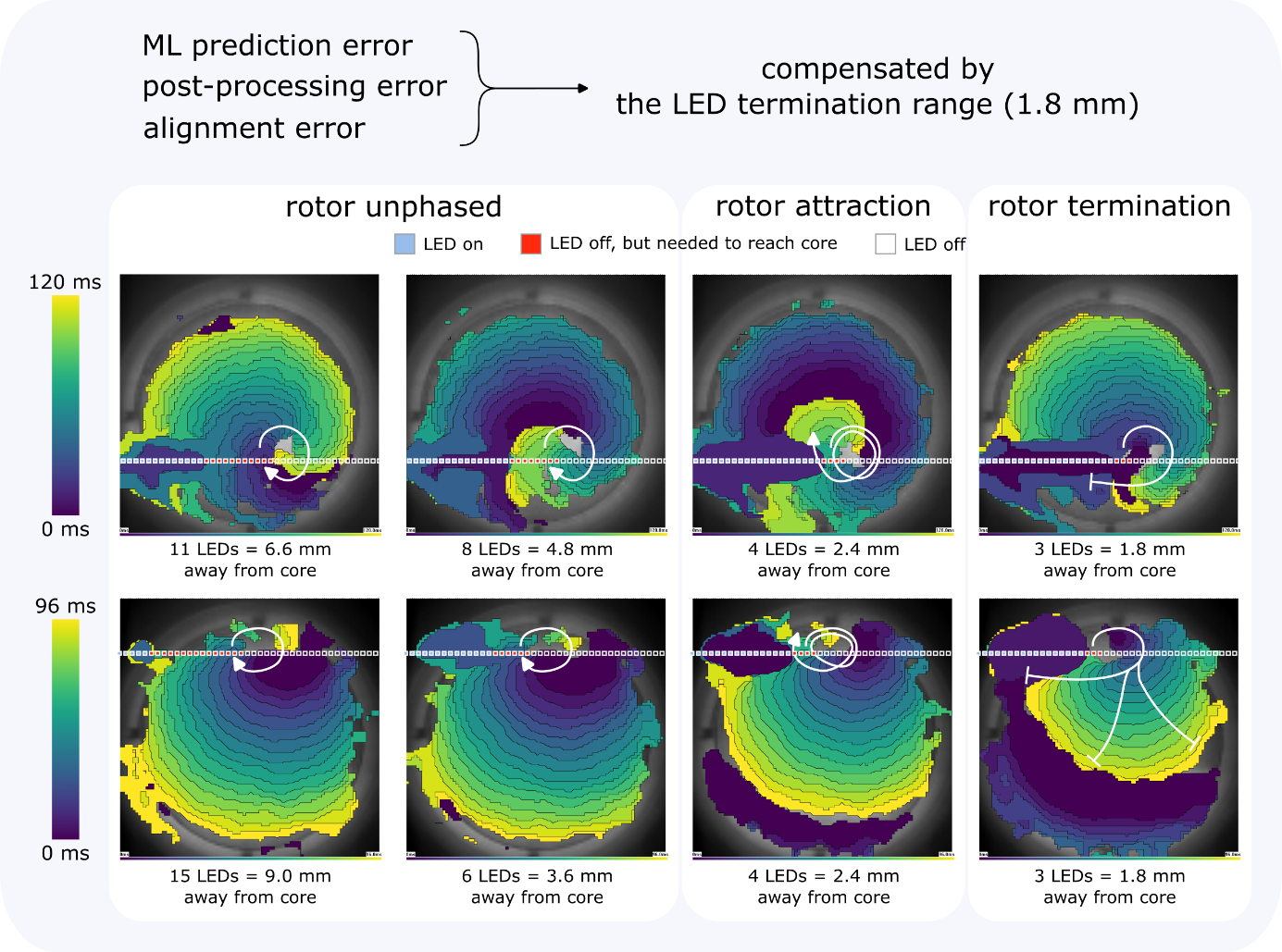


**Figure S14.** Error tolerance determined by the LED termination range. System-level errors are primarily governed by the spatial termination range of the LEDs, defined as the maximum distance from the phase singularity at which illumination can terminate the rotor. When illumination is applied at distances greater than four LEDs (pitch = 0.6 mm), the rotor remains unaffected. At a distance of four LEDs, transient rotor attraction occurs during 500 ms of illumination, with full recovery once the light is switched off. At distances of three LEDs or fewer, the rotor is consistently terminated (n=3), corresponding to an overall error tolerance of 1.8 mm.


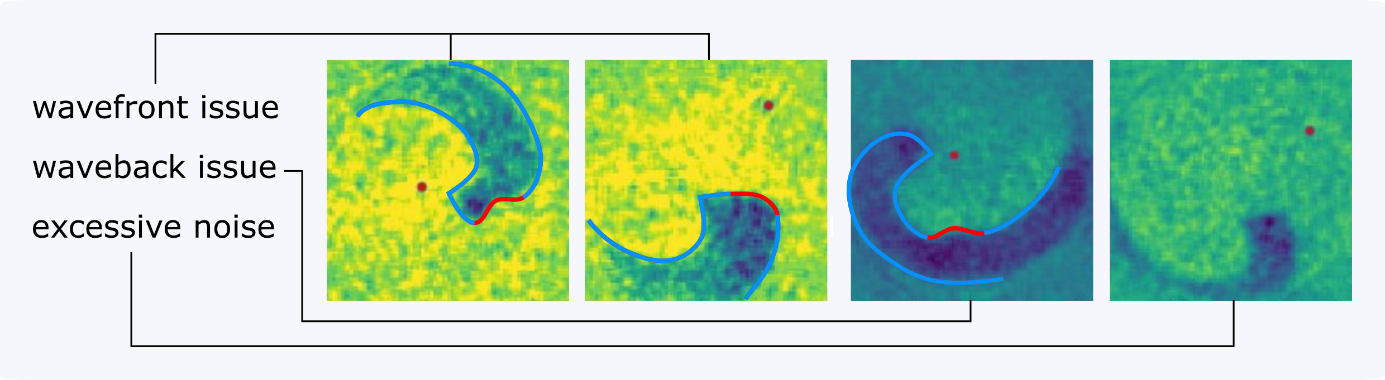
**Figure S15.** Most common failure patterns when multiple tries where needed to terminate the reentry were wavefront and back issues, as well as excessive noise.


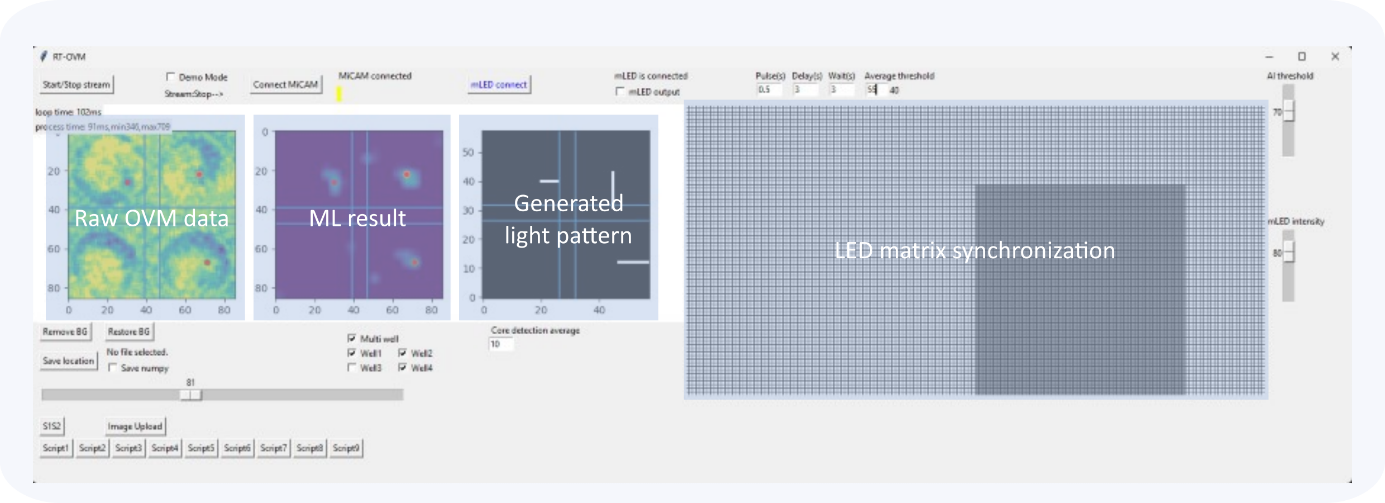


**Figure S16.** Graphical user interface (UI) displaying all control options, including thresholds, quadrant-specific CNN activation, waiting time, and other parameters.
