# Supplementary figures and images for: Smart Optogenetics for Real‐Time Automated Control of Cardiac Electrical Activity
Source: Adv Sci (Weinh). 2026 Feb 13;13(20):e22759. doi: 10.1002/advs.202522759 (PMC13067851; doi:10.1002/advs.202522759)

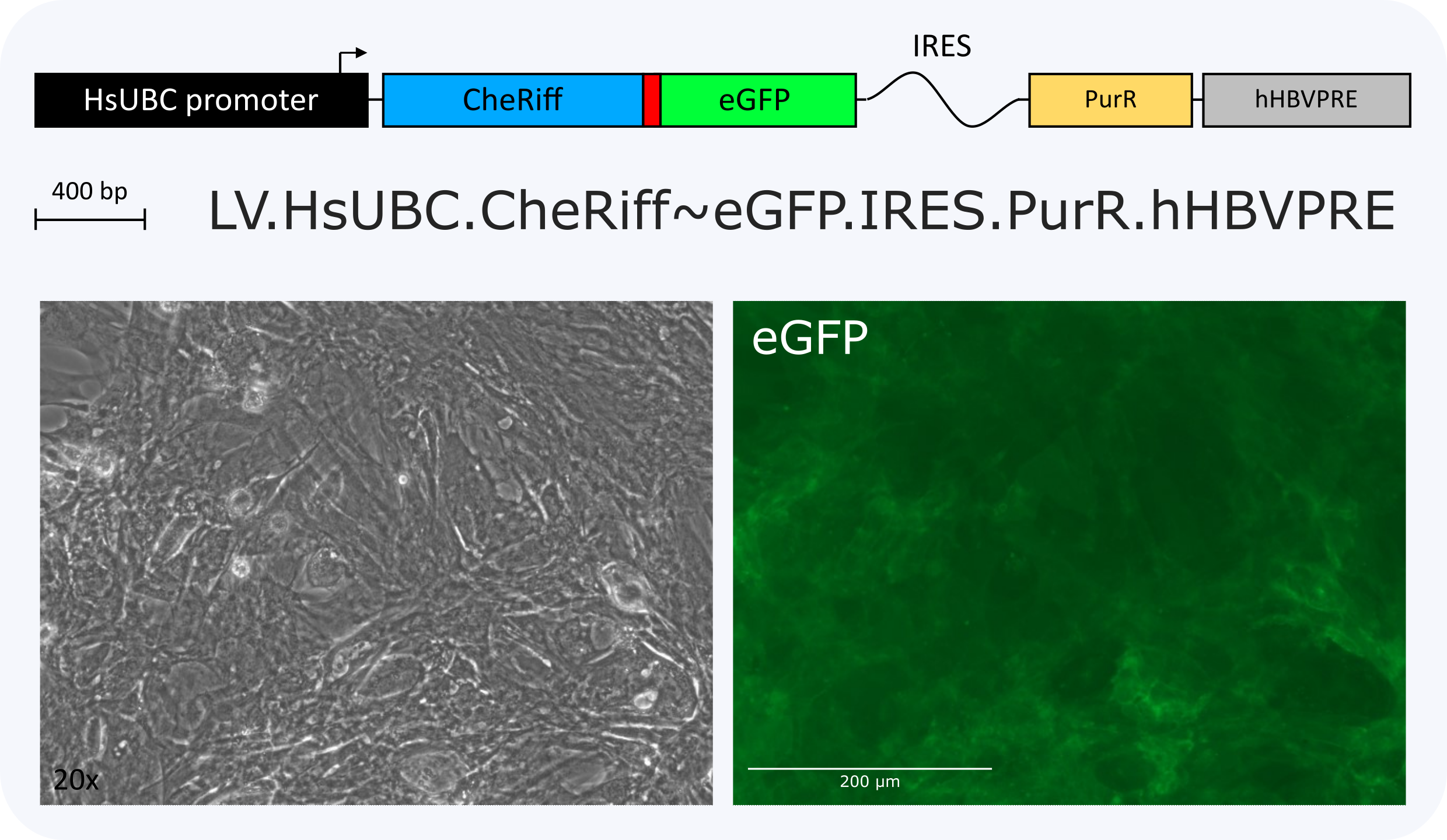

Supplement: Supplementary file 2 — Supporting File 2: advs74173‐sup‐0002‐FigureS1‐S16.zip. [file ADVS-13-e22759-s001.zip › OptoAI_FigS1.png]

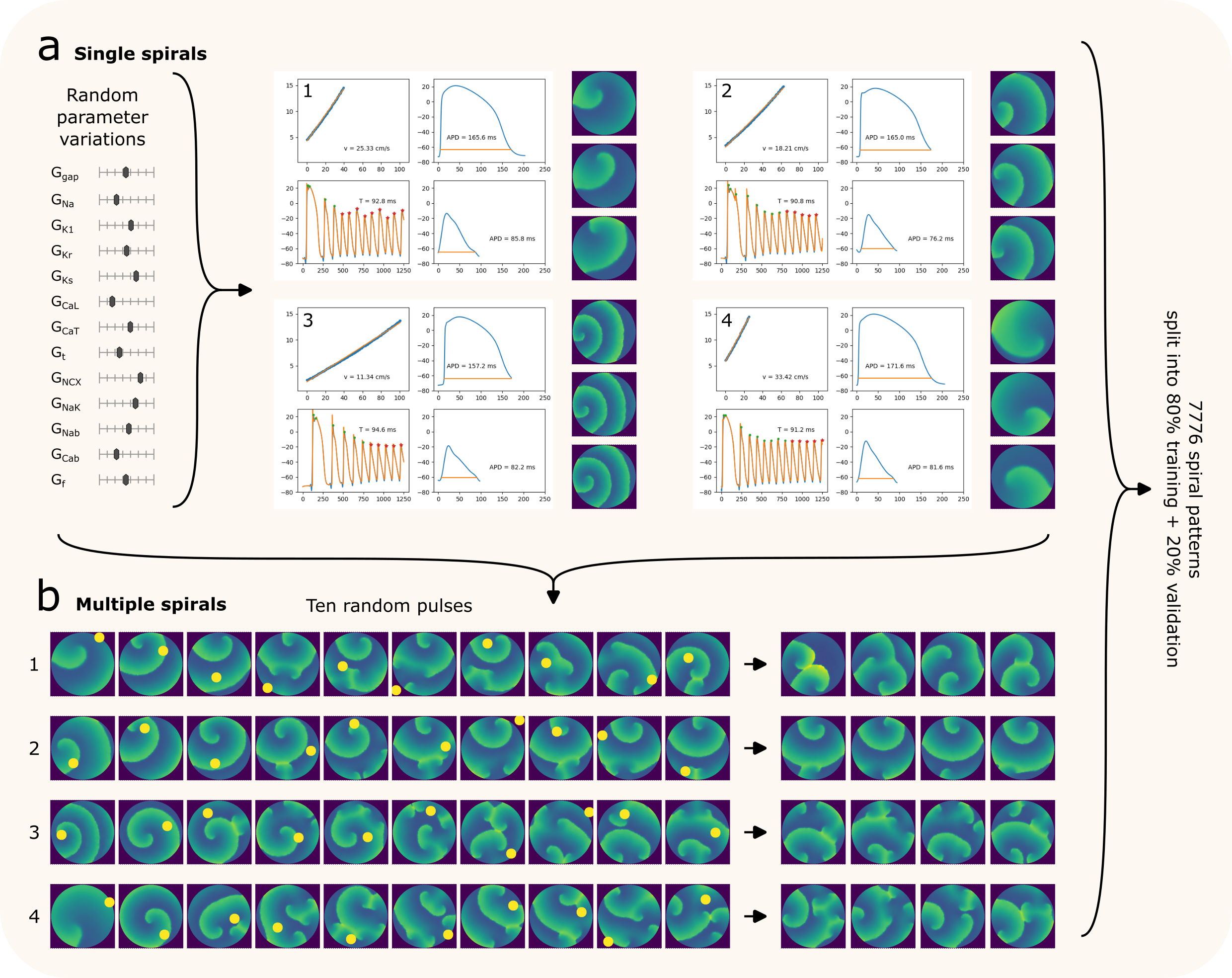

Supplement: Supplementary file 2 — Supporting File 2: advs74173‐sup‐0002‐FigureS1‐S16.zip. [file ADVS-13-e22759-s001.zip › OptoAI_FigS10.png]

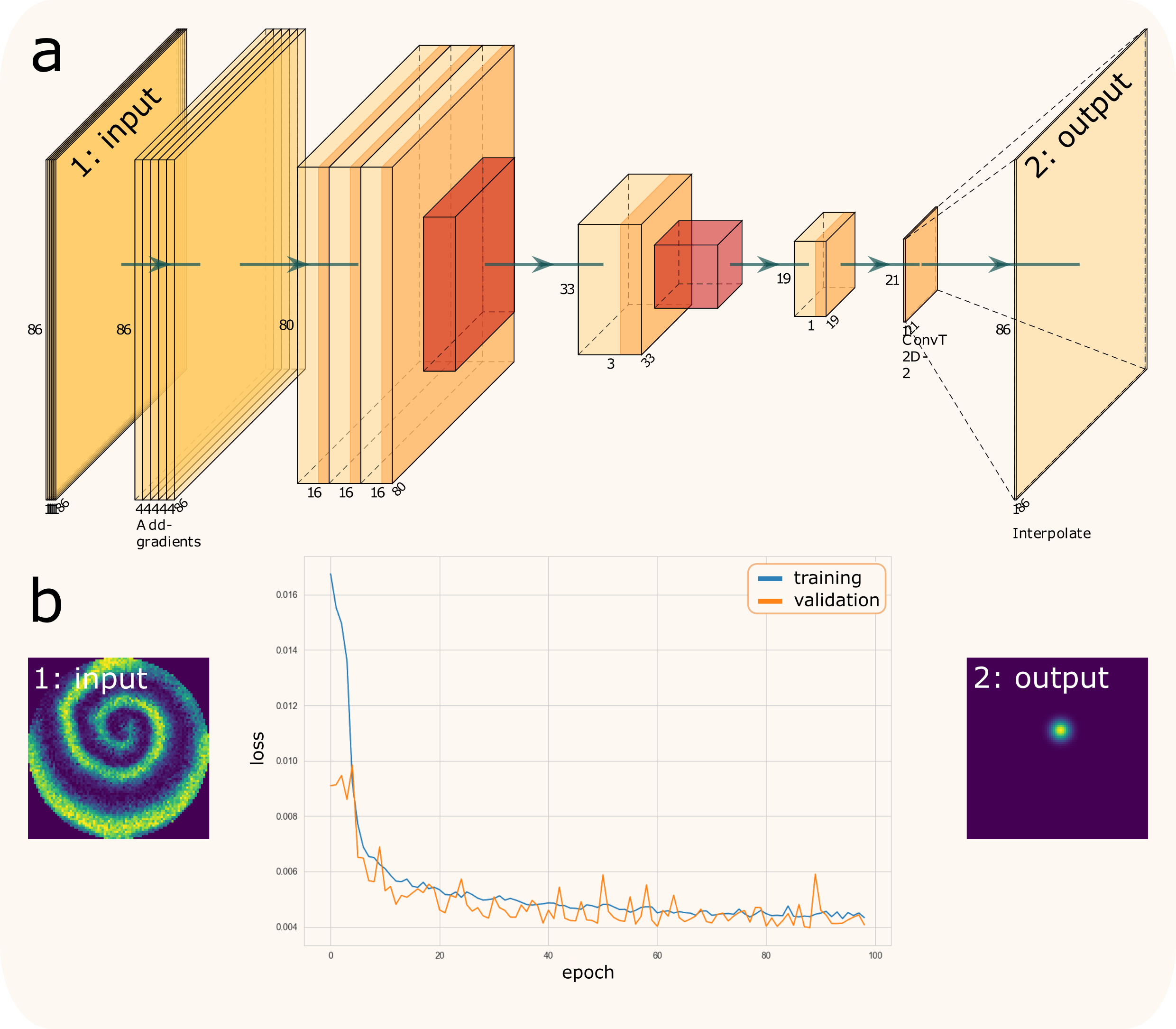

Supplement: Supplementary file 2 — Supporting File 2: advs74173‐sup‐0002‐FigureS1‐S16.zip. [file ADVS-13-e22759-s001.zip › OptoAI_FigS11.png]

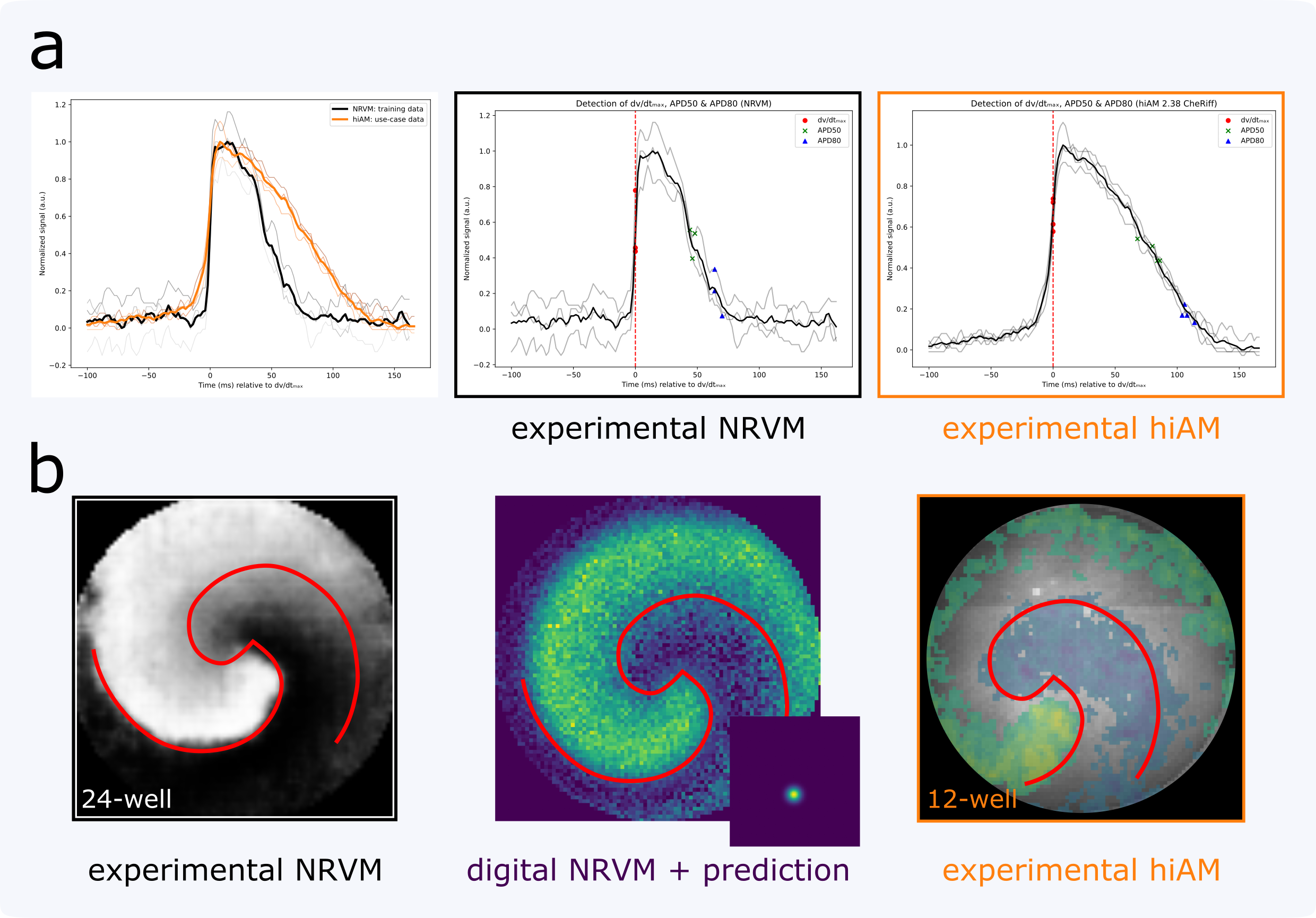

Supplement: Supplementary file 2 — Supporting File 2: advs74173‐sup‐0002‐FigureS1‐S16.zip. [file ADVS-13-e22759-s001.zip › OptoAI_FigS12.png]

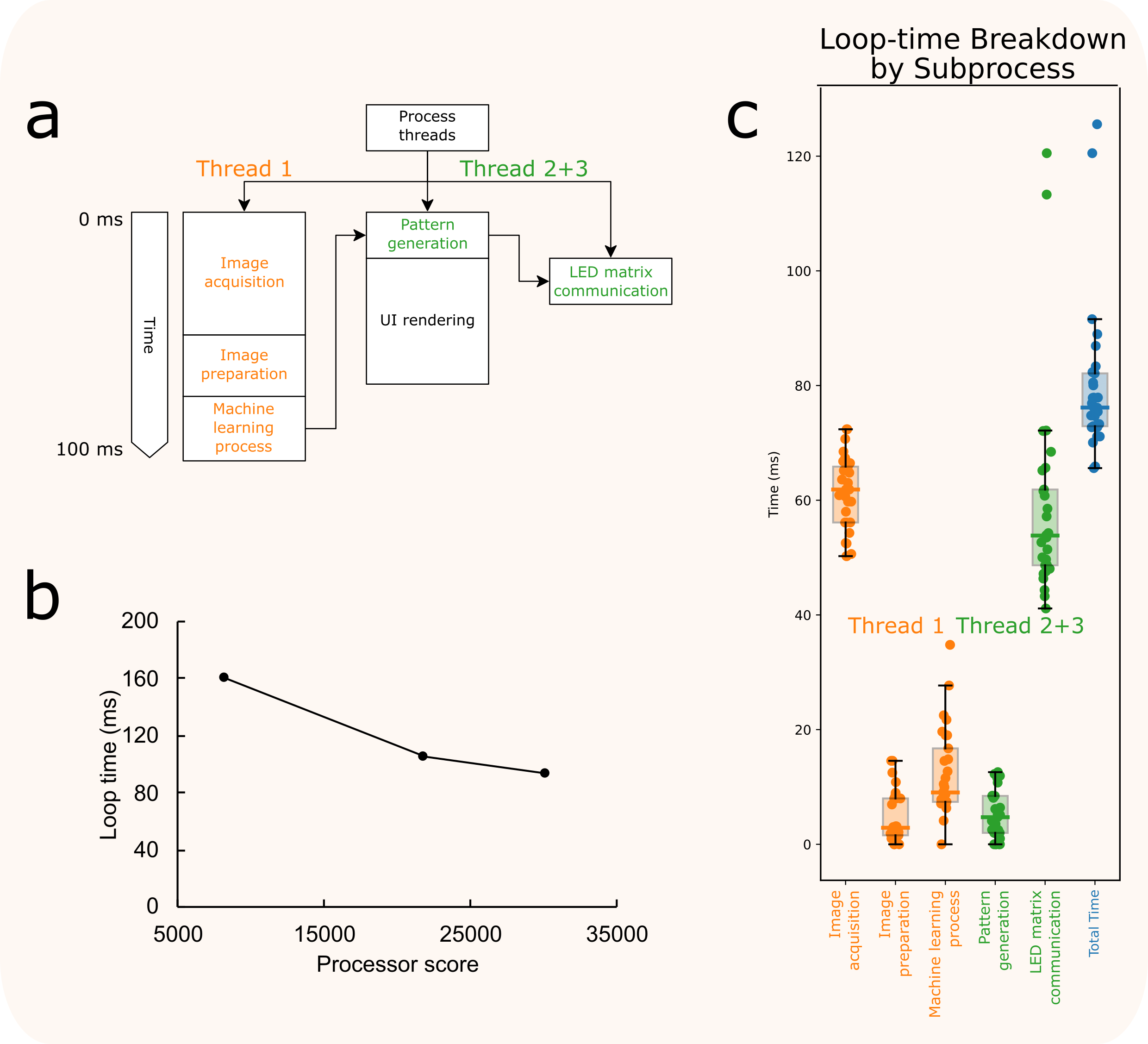

Supplement: Supplementary file 2 — Supporting File 2: advs74173‐sup‐0002‐FigureS1‐S16.zip. [file ADVS-13-e22759-s001.zip › OptoAI_FigS13.png]

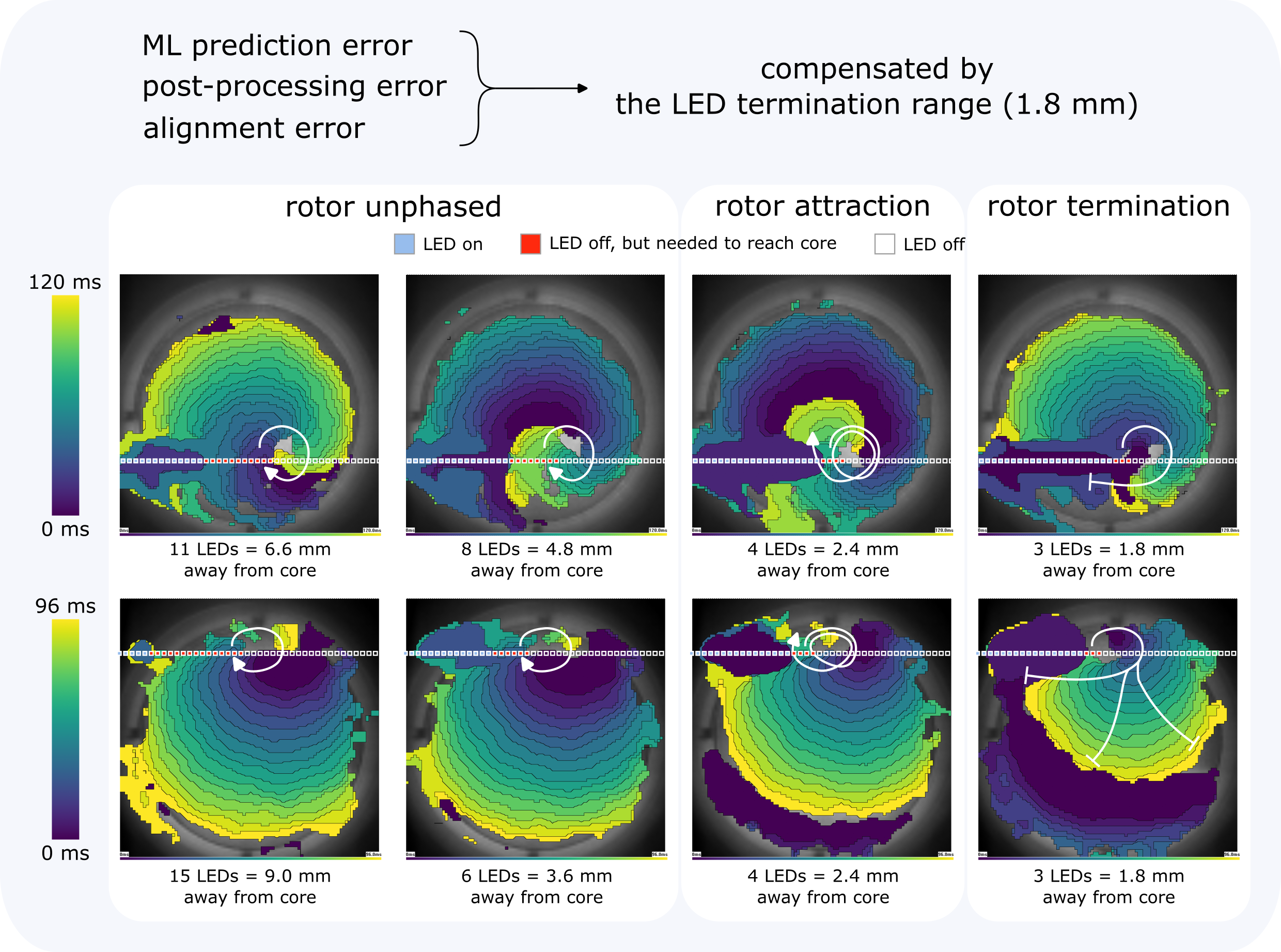

Supplement: Supplementary file 2 — Supporting File 2: advs74173‐sup‐0002‐FigureS1‐S16.zip. [file ADVS-13-e22759-s001.zip › OptoAI_FigS14.png]

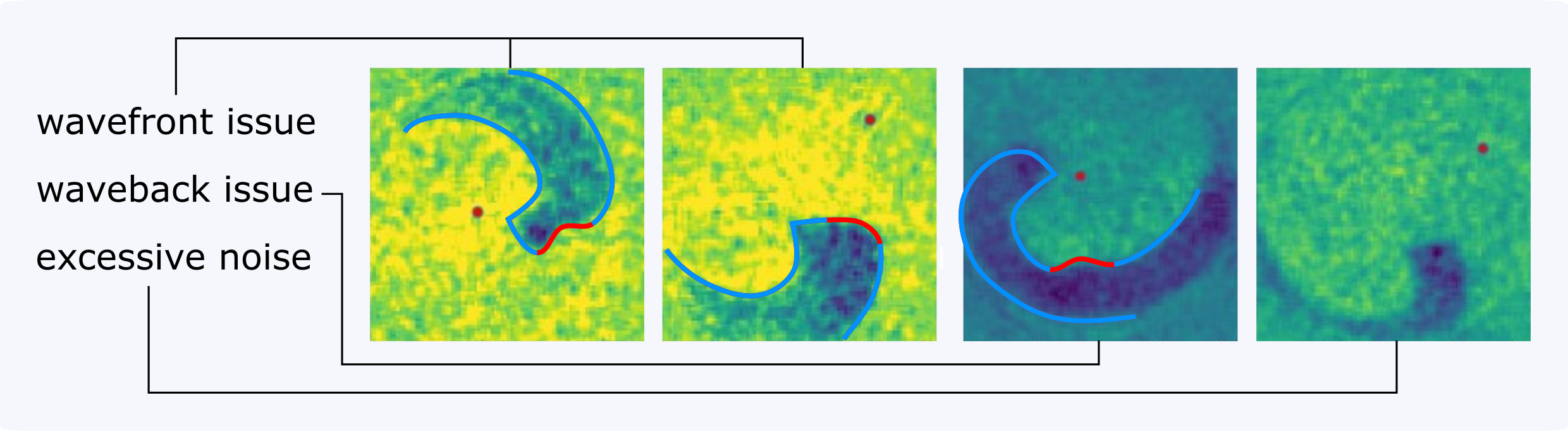

Supplement: Supplementary file 2 — Supporting File 2: advs74173‐sup‐0002‐FigureS1‐S16.zip. [file ADVS-13-e22759-s001.zip › OptoAI_FigS15.png]

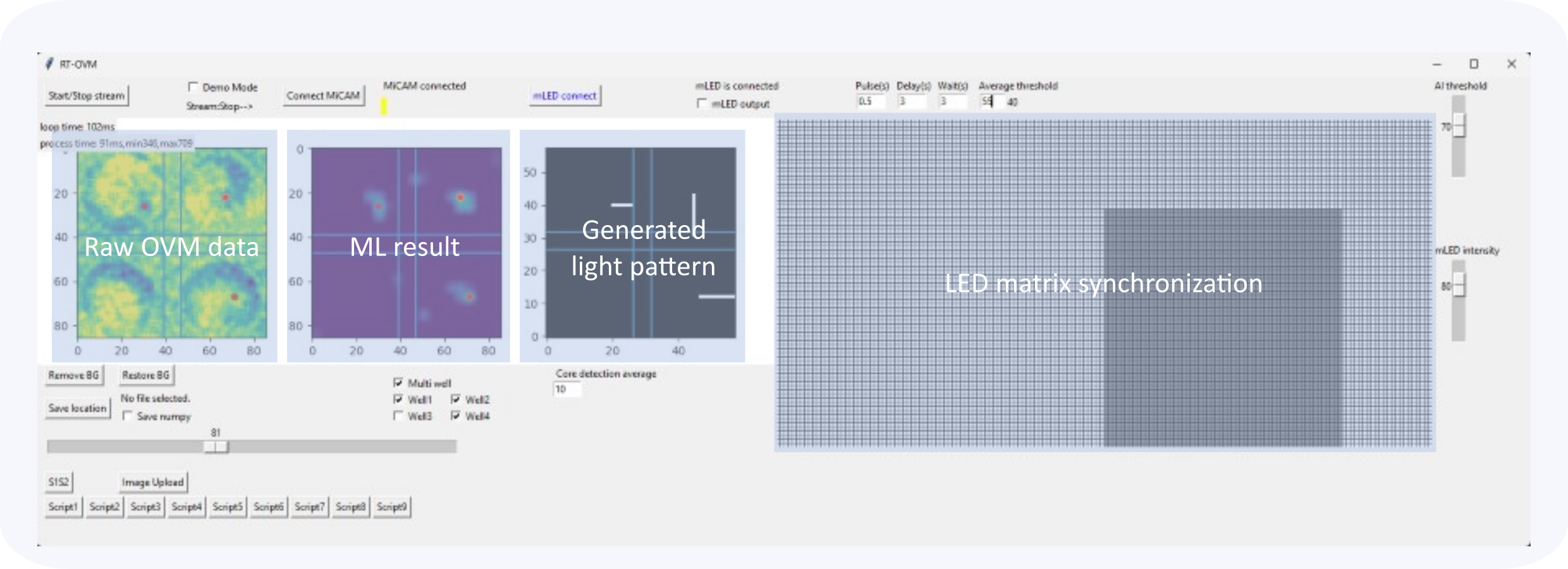

Supplement: Supplementary file 2 — Supporting File 2: advs74173‐sup‐0002‐FigureS1‐S16.zip. [file ADVS-13-e22759-s001.zip › OptoAI_FigS16.png]

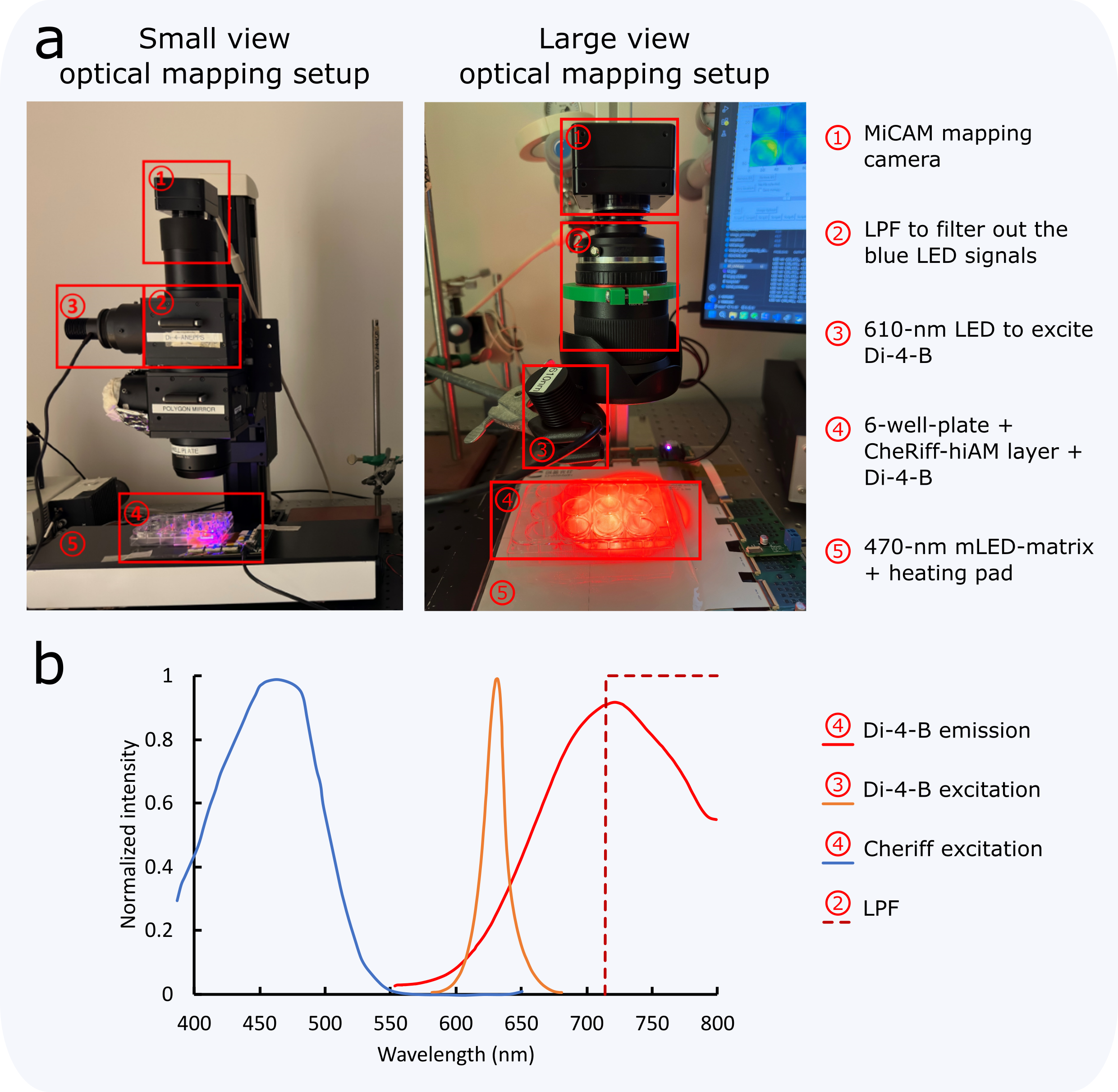

Supplement: Supplementary file 2 — Supporting File 2: advs74173‐sup‐0002‐FigureS1‐S16.zip. [file ADVS-13-e22759-s001.zip › OptoAI_FigS2.png]

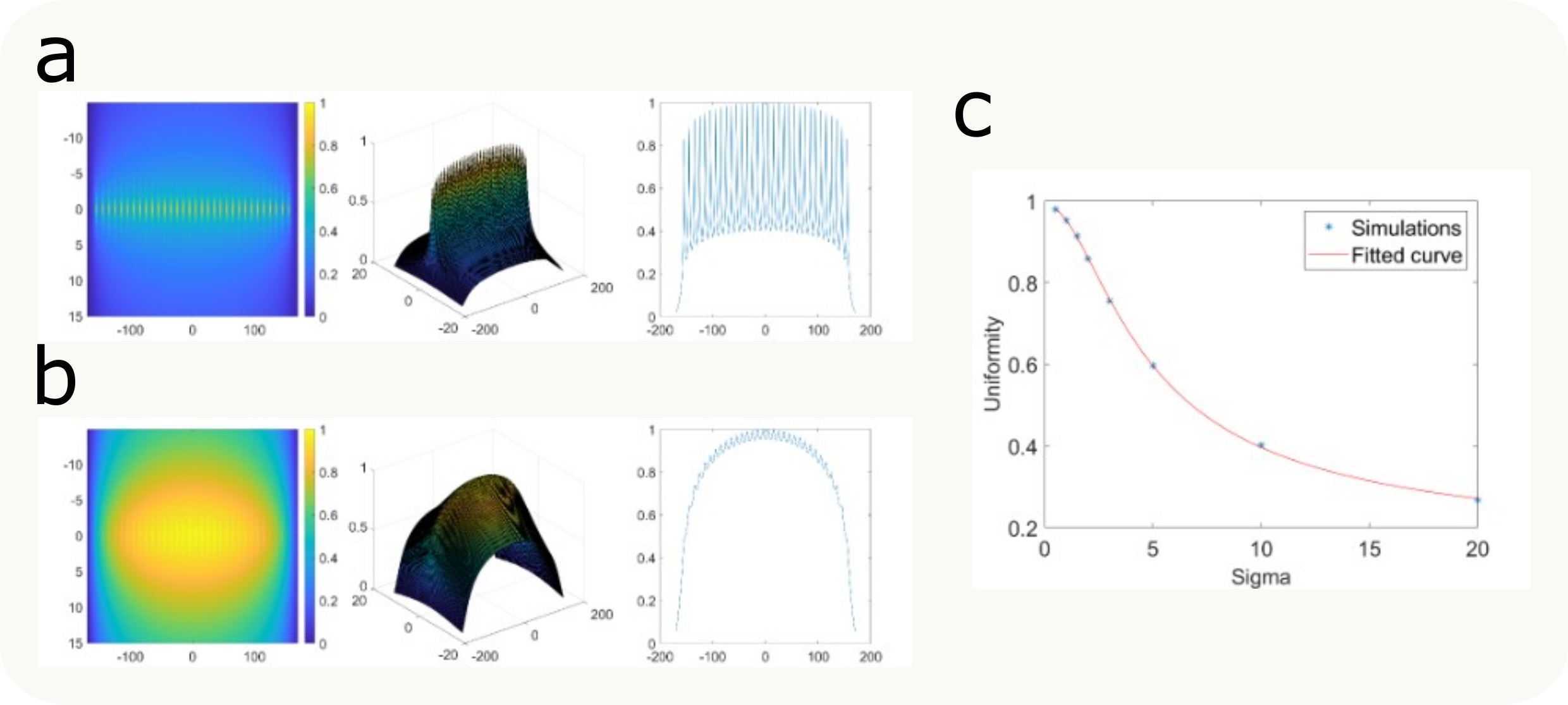

Supplement: Supplementary file 2 — Supporting File 2: advs74173‐sup‐0002‐FigureS1‐S16.zip. [file ADVS-13-e22759-s001.zip › OptoAI_FigS3.png]

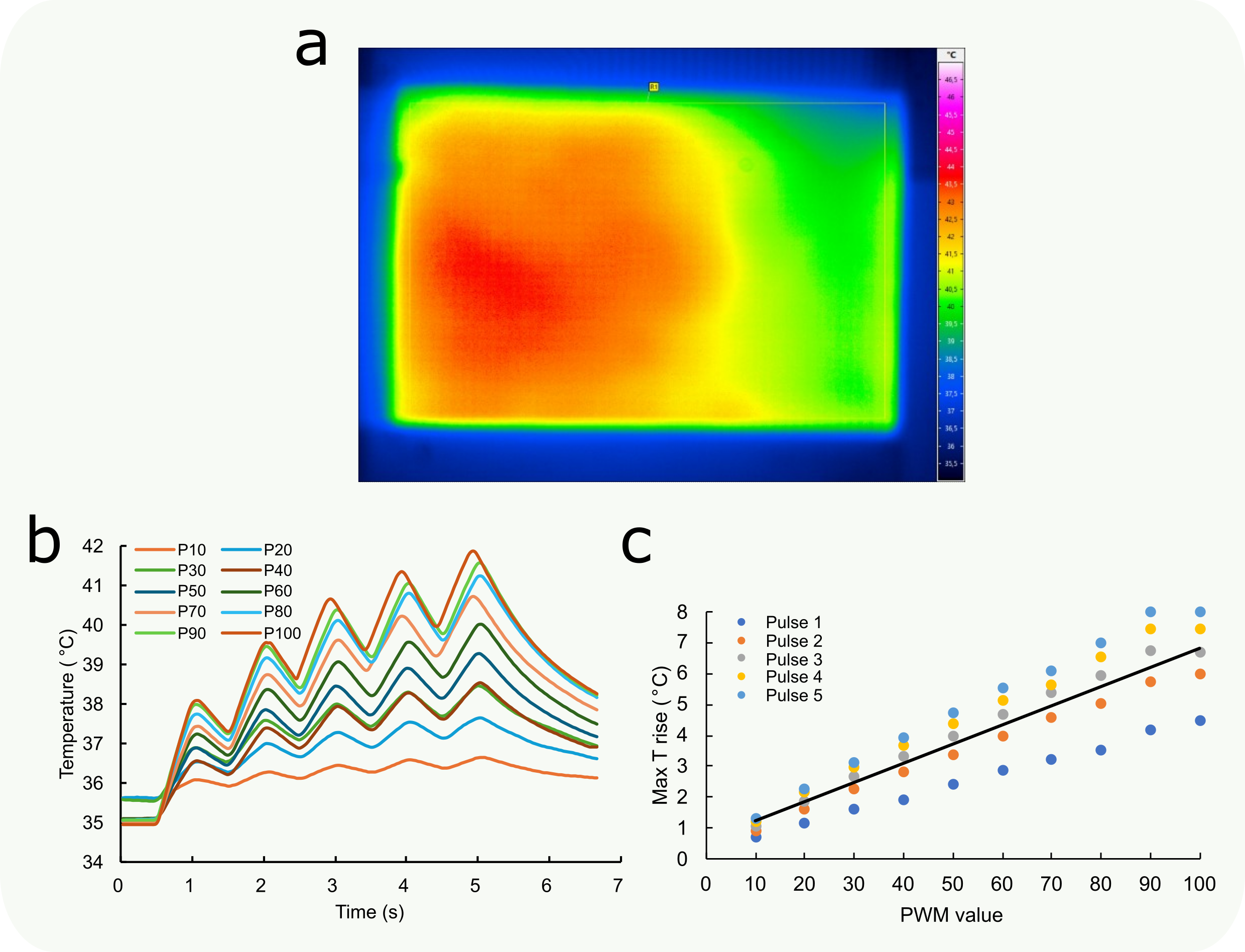

Supplement: Supplementary file 2 — Supporting File 2: advs74173‐sup‐0002‐FigureS1‐S16.zip. [file ADVS-13-e22759-s001.zip › OptoAI_FigS4.png]

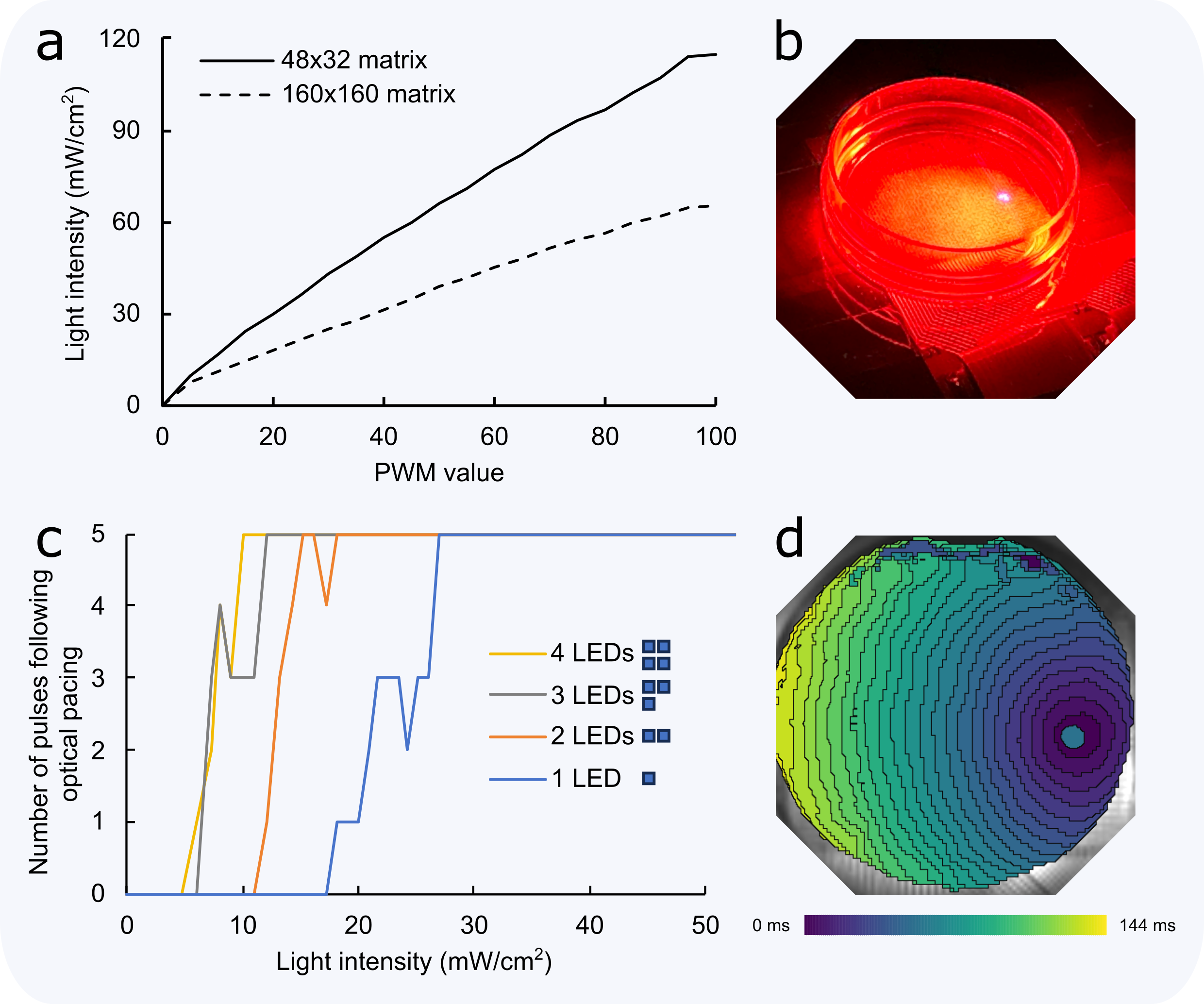

Supplement: Supplementary file 2 — Supporting File 2: advs74173‐sup‐0002‐FigureS1‐S16.zip. [file ADVS-13-e22759-s001.zip › OptoAI_FigS5.png]

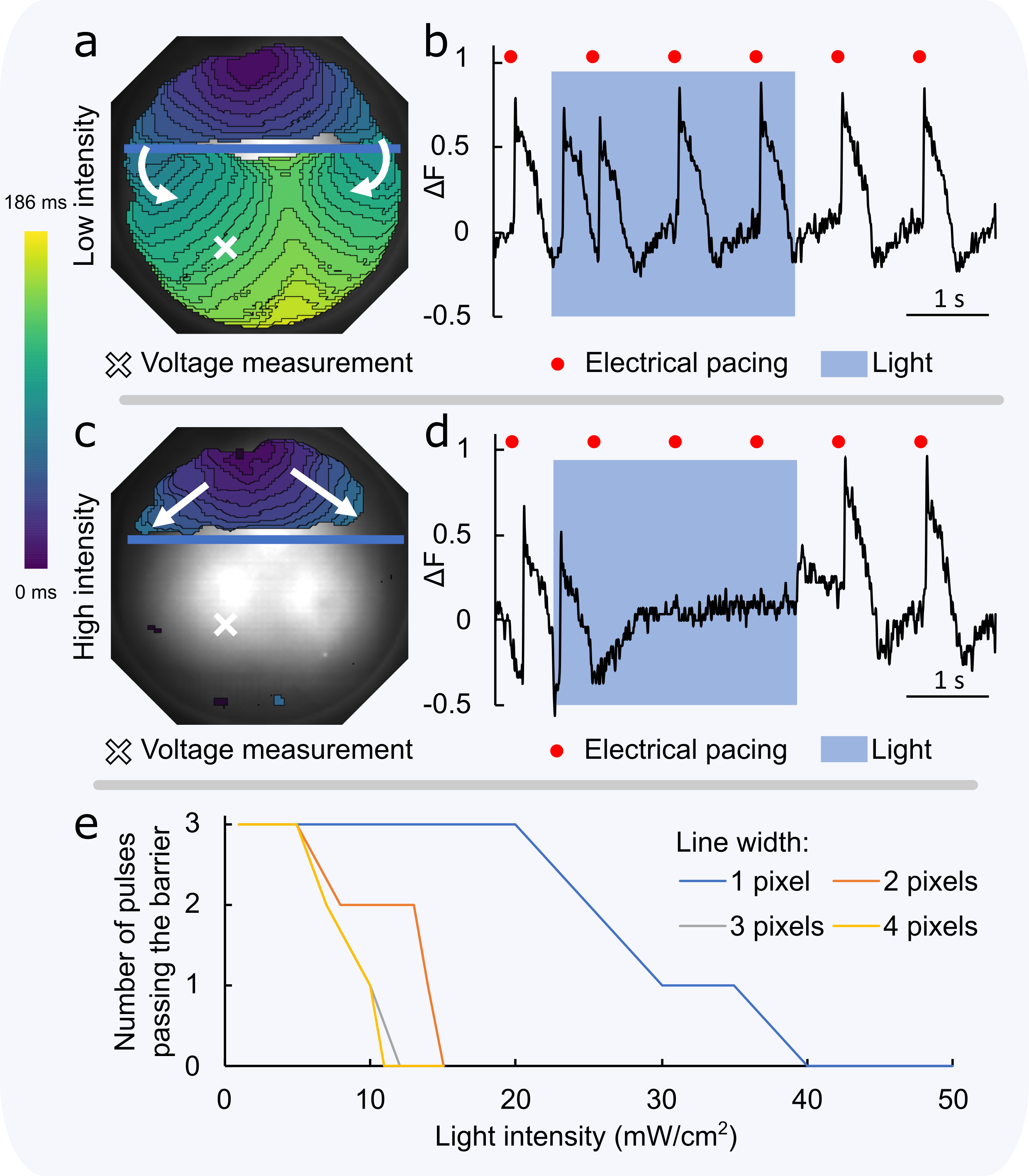

Supplement: Supplementary file 2 — Supporting File 2: advs74173‐sup‐0002‐FigureS1‐S16.zip. [file ADVS-13-e22759-s001.zip › OptoAI_FigS6.png]

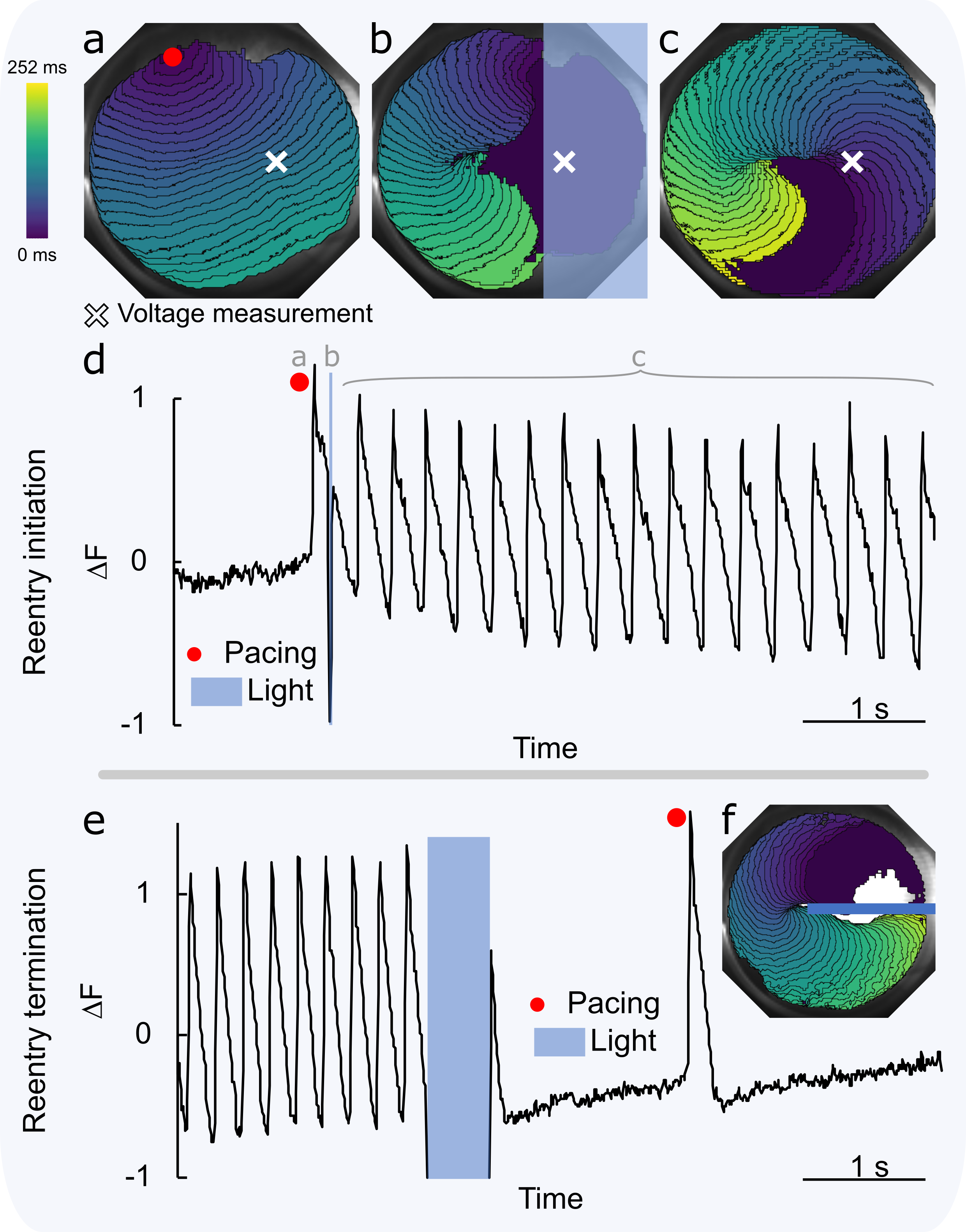

Supplement: Supplementary file 2 — Supporting File 2: advs74173‐sup‐0002‐FigureS1‐S16.zip. [file ADVS-13-e22759-s001.zip › OptoAI_FigS7.png]

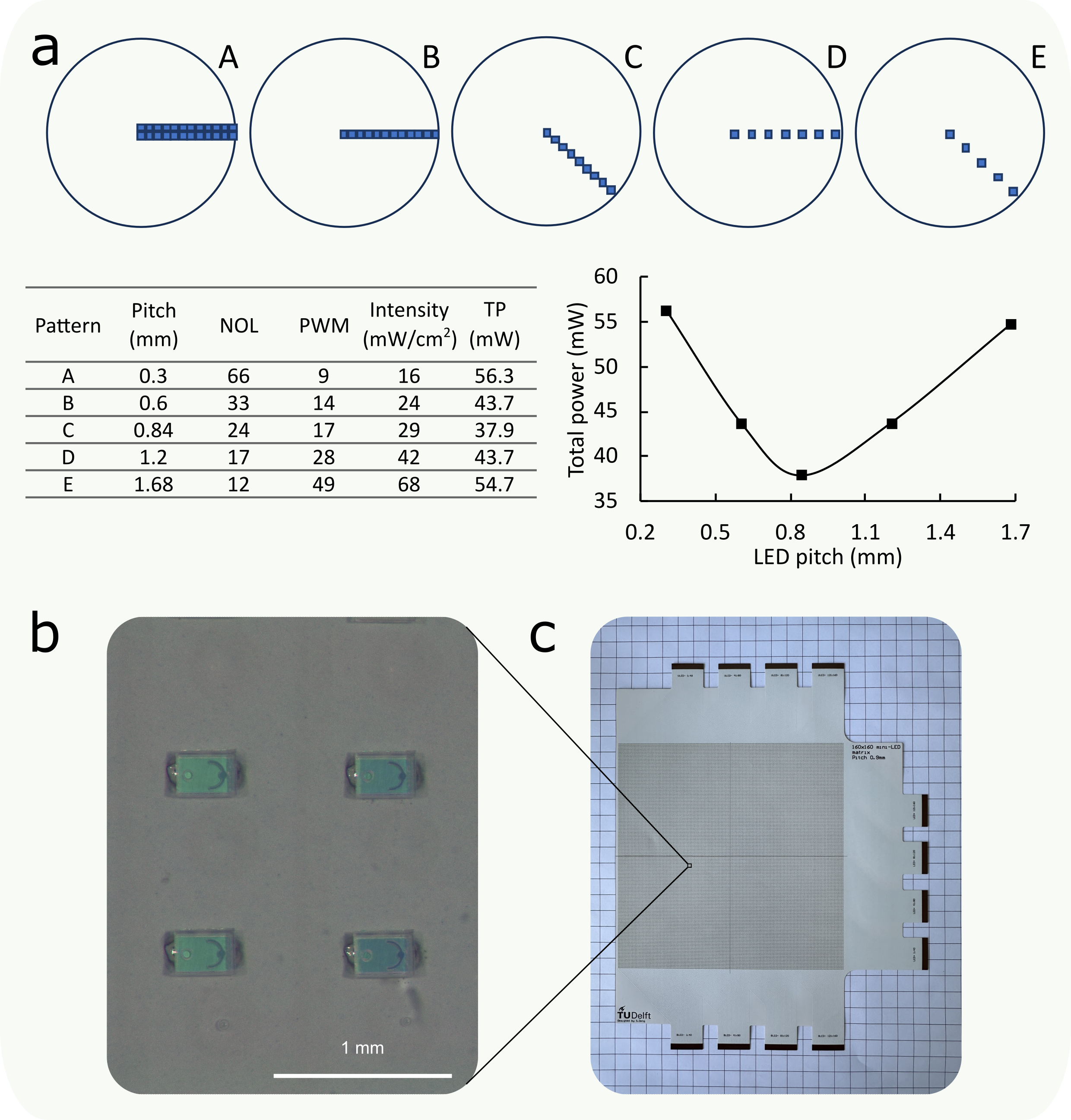

Supplement: Supplementary file 2 — Supporting File 2: advs74173‐sup‐0002‐FigureS1‐S16.zip. [file ADVS-13-e22759-s001.zip › OptoAI_FigS8.png]

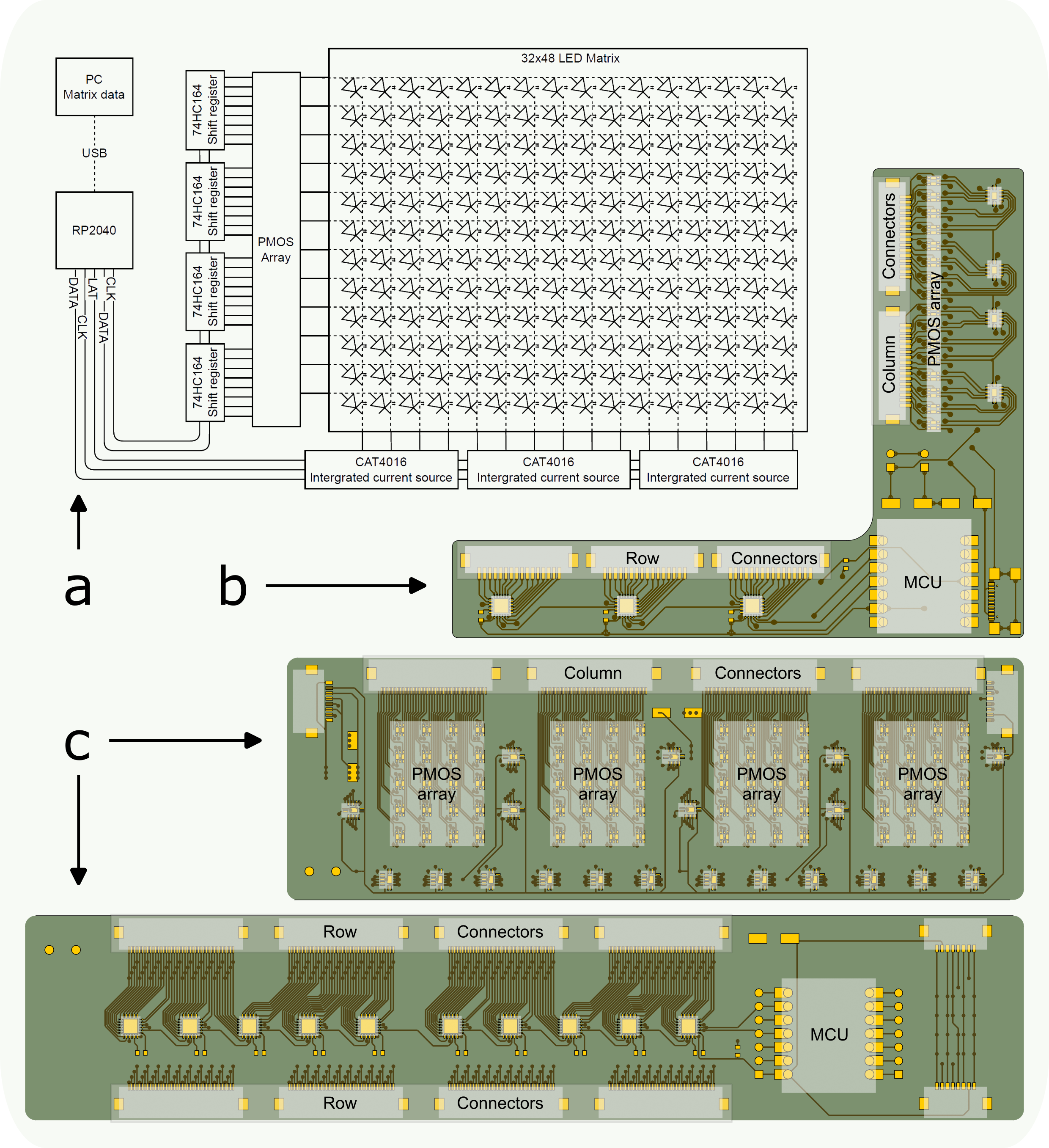

Supplement: Supplementary file 2 — Supporting File 2: advs74173‐sup‐0002‐FigureS1‐S16.zip. [file ADVS-13-e22759-s001.zip › OptoAI_FigS9.png]
